# Phlogopite-pargasite coexistence in an oxygen reduced spinel-peridotite ambient

**DOI:** 10.1038/s41598-021-90844-w

**Published:** 2021-06-04

**Authors:** Costanza Bonadiman, Valentina Brombin, Giovanni B. Andreozzi, Piera Benna, Massimo Coltorti, Nadia Curetti, Barbara Faccini, Marcello Merli, Beatrice Pelorosso, Vincenzo Stagno, Magdala Tesauro, Alessandro Pavese

**Affiliations:** 1 Dipartimento di Fisica e Scienze della Terra, Università degli Studi di Ferrara, Via Saragat 1, 44122 Ferrara, Italy; 2 Istituto di Geologia Ambientale e Geoingegneria del Consiglio Nazionale delle Ricerche (IGAG-CNR), Via Salaria km 29, 300, 00015 Montelibretti, Italy; 3 Istituto di Geoscienze e Georisorse del Consiglio Nazionale delle Ricerche (CNR-IGG-CNR), Via G. Moruzzi 1, 56124 Pisa, Italy; 4 Dipartimento di Scienze della Terra, Sapienza Università di Roma, Piazzale Aldo Moro 5, 00185 Rome, Italy; 5 Dipartimento di Scienze della Terra, Università degli Studi di Torino, Via Valperga Caluso 35, 10100 Turin, Italy; 6 Dipartimento di Scienze della Terra e del Mare (DiSTeM), Università di Palermo, Via Archirafi 36, 90123 Palermo, Italy; 7 Dipartimento di Matematica e Geoscienze, Università di Trieste, Via Weiss 2, 34128 Trieste, Italy; 8 Department of Earth Sciences, Utrecht University, Princetonlaan 8a, Utrecht, 3584 CB The Netherlands

**Keywords:** Geochemistry, Mineralogy, Petrology

## Abstract

The occurrence of phlogopite and amphibole in mantle ultramafic rocks is widely accepted as the modal effect of metasomatism in the upper mantle. However, their simultaneous formation during metasomatic events and the related sub-solidus equilibrium with the peridotite has not been extensively studied. In this work, we discuss the geochemical conditions at which the pargasite-phlogopite assemblage becomes stable, through the investigation of two mantle xenoliths from Mount Leura (Victoria State, Australia) that bear phlogopite and the phlogopite + amphibole (pargasite) pair disseminated in a harzburgite matrix. Combining a mineralogical study and thermodynamic modelling, we predict that the *P*–*T locus* of the equilibrium reaction pargasite + forsterite = Na-phlogopite + 2 diopside + spinel, over the range 1.3–3.0 GPa/540–1500 K, yields a negative Clapeyron slope of -0.003 GPa K^–1^ (on average). The intersection of the *P–T locus* of supposed equilibrium with the new mantle geotherm calculated in this work allowed us to state that the Mount Leura xenoliths achieved equilibrium at 2.3 GPa /1190 K, that represents a plausible depth of ~ 70 km. Metasomatic K-Na-OH rich fluids stabilize hydrous phases. This has been modelled by the following equilibrium equation: 2 (K,Na)-phlogopite + forsterite = 7/2 enstatite + spinel + fluid (components: Na_2_O,K_2_O,H_2_O). Using quantum-mechanics, semi-empirical potentials, lattice dynamics and observed thermo-elastic data, we concluded that K-Na-OH rich fluids are not effective metasomatic agents to convey alkali species across the upper mantle, as the fluids are highly reactive with the ultramafic system and favour the rapid formation of phlogopite and amphibole. In addition, oxygen fugacity estimates of the Mount Leura mantle xenoliths [Δ(FMQ) = –1.97 ± 0.35; –1.83 ± 0.36] indicate a more reducing mantle environment than what is expected from the occurrence of phlogopite and amphibole in spinel-bearing peridotites. This is accounted for by our model of full molecular dissociation of the fluid and incorporation of the O-H-K-Na species into (OH)-K-Na-bearing mineral phases (phlogopite and amphibole), that leads to a peridotite metasomatized ambient characterized by reduced oxygen fugacity.

## Introduction

Phlogopite [KMg_3_AlSi_3_O_10_(OH)_2_] is a common hydrous mineral that occurs in ultramafic-alkaline igneous rocks, including kimberlites, aillikites, orangeites, and carbonatites^1–4^. This mineral is frequently observed in cratonic mantle (garnet- and spinel- bearing) xenoliths hosted in rocks of the diatremic association, such as kimberlites, and occurs mainly along with amphibole, another hydrous mineral, in ultramafic rocks formed by cumulate processes^5,6^, in peridotite massifs^7^ and in off-craton mantle spinel-bearing xenoliths^8–10^. In particular, phlogopite in lherzolites and harzburgites from off-craton mantle xenoliths that achieved equilibrium in the spinel stability field is scarce (rarely > 1%^9^), whereas it becomes modally relevant in pyroxenite and wehrlite lithotypes^6,11^. The presence of phlogopite and/or amphibole in an anhydrous-dominated ultramafic system is widely accepted to mark the occurrence of “modal mantle metasomatism”^12^, as phlogopite and amphibole crystallization is mainly due to the interaction between metasomatic K(Na)-OH rich “fluids/melts” (e.g., alkaline mafic melts) and variously depleted peridotites, in a porous flow regime^13–15^. Such metasomatic reactions have extensively modified the sub-continental lithospheric mantle (SCLM) since the Archean^16–22^.

The metasomatic processes take place even though K-Na-OH are chemical species showing modest compatibility or even incompatibility with the peridotite minerals (i.e., olivine, orthopyroxene, clinopyroxene, spinel or garnet) in geochemical reactions. In the petrological system of the upper mantle consisting of a source-melt-residuum, the mineral/melt partitioning of K(Na)-OH shifts towards the melt, i.e., D = C_mineral_/C_melt_ < 0.1 (where D = distribution coefficient; C = concentration). Only Na is considered a *mildly* incompatible element in pyroxenes, yielding a D of ~ 0.1 at < 1.5 GPa, which increases with higher pressure conditions^23^.

Phlogopite (and amphibole) melting is often invoked to account for a high potassium content in mantle-derived melts^24–26^. For instance, PIC (Phlogopite-Ilmenite-Clinopyroxene) and MARID (Mica-Amphibole-Rutile-Ilmenite-Diopside) mantle assemblages are supposed to account for the origin of alkali rich mafic–ultramafic magmas^20,27^. Conversely, the preservation of phlogopite and amphibole during low degree partial melting induces significant depletion of potassium in the derived melts^28^.

Many experimental studies on natural peridotite deal with either phlogopite^15^ or amphibole^29^ stability and ascribe the occurrence of these two minerals to a recent metasomatism^6^. Such studies are generally focused on understanding the relationship between minerals and magma source region^24,29,30^, as well as unravelling the nature of the metasomatic fluids/melts in the upper mantle^6,7,13,21,31^. Although the stability of the hydrous phases affects both mantle solidus and magma genesis^24,30^, the sub-solidus equilibrium of the phlogopite + amphibole system with the anhydrous peridotite minerals has attracted moderate attention and the experimental confirmation of this paragenesis in ultramafic systems is only provided by solidus reactions involving fluids (see Safonov et al.^15^ for a review).

In this work, we explored the physical–chemical mantle conditions (*P*, *T*, ƒO_2_) at which the phlogopite + amphibole system stabilized. In particular, we investigated two mantle xenoliths from Mount Leura (Victoria State, southeast Australia) in which phlogopite (LE7_[phl]_) and phlogopite + amphibole (LE5_[phl+amph]_) occur. They are thought to be products of an early metasomatism, which subsequently underwent textural (re) equilibration with primary anhydrous minerals. We used laboratory characterizations complemented by theoretical modelling to determine: (i) the *P*–*T* conditions at the formation of the peridotite + phlogopite + amphibole assemblage; (ii) the stability conditions of potential metasomatic fluids in a K-Na-OH peridotite ambient; (iii) the related redox conditions driving the reactions.

## Sample description and petrography

The two samples were collected in Cenozoic basanites from Mount Leura, one of the richest xenolith sites of the Newer Volcanic Province (4.5 Ma-5000 B.P), in western Victoria state, southeast Australia^32,33^. Peridotite are the predominant lithotype of xenoliths in the Newer Volcanic Province. In particular, spinel lherzolites can be found in 28 localities (e.g., Mounts Porndon, Leura, Noorat, etc.^32,34^). These xenoliths are fragments from the shallow portion of the continental lithospheric mantle, carried to the surface by a relatively recent activity of the Newer Volcanic Province^32^, as a late effect of the continental extension related to the breakup of Gondwana and opening of the Tasman Sea^32,34^.

Both xenoliths (LE7_[phl]_ and LE5_[phl+amph]_) are sub-rounded in shape, ~ 7 cm in diameter, apparently free of host magma infiltrations, though infiltrations of exotic (e.g., magmatic) fluids/melts cannot be excluded.

Their mineral phase proportions were estimated by mass balance calculations between bulk rock and mineral phase compositions, using a non-weighted least-squares regression of the major element oxides (Table 1).

**Table 1:** Bulk rock major (wt%) and minor (ppm) element compositions; average phase proportions (%); geothermal and oxygen fugacity estimates of the Mt. Leura (LE) peridotite xenoliths. Average temperature (*T*) and oxygen fugacity (Δlog*f*O_2_) for LE harzburgites are calculated at 2 GPa. Estimated temperatures are from the two-pyroxene geothermometer of Brey and Köhler^36^, and olivine-spinel geothermometers of Ballhaus et al.^37^ and Jianping et al.^38^. Δlog*f*O_2_ (FMQ) is calculated from the method of Wood^39^ using Brey and Köhler^36^ temperature and it is reported as difference (Δ) with respect to Fayalite-Magnetite-Quartz (FMQ) buffer^40^. Uncertainties are reported.

Sample	LE7_[phl]_	LE5_[phl+amph]_
	Harzburgite	Harzburgite
SiO_2_	44.22	44.82
TiO_2_	0.02	0.06
Al_2_O_3_	0.21	0.69
FeO_tot_	8.39	9.78
MnO	0.11	0.14
MgO	45.94	42.91
CaO	0.48	0.91
Na_2_O	0.00	0.08
K_2_O	0.01	0.06
P_2_O_5_	0.00	0.02
LOI	0.63	0.53
Total	100	100
Mg#	91.56	89.68
Cr	2051	2900
Ni	2529	2191
T_Opx-Cpx_ (K)	1313 ± 15	1175 ± 32
T_Ol-Sp_ (K)	1280 ± 100	1151 ± 83
Δlog*f*O_2_ (FMQ)	–1.83 ± 0.36	–1.97 ± 0.65
Olivine	73	69
Orthopyroxene	21	24
Clinopyroxene	3.7	4.0
Spinel	1.0	1.0
Phlogopite	1.9	1.2
Amphibole	–	1.4

Mg# = 100 × Mg/(Mg + Fe)_mol_.

Given that the xenoliths under study contain a spinel peridotite assemblage (olivine + clinopyroxene + orthopyroxene + spinel) with less than 5% clinopyroxene (Table 1), they are classified as harzburgites (according to the IUGS scheme^35^). Both harzburgites bear observable amounts of phlogopite: 1.9% in LE7_[phl]_ and 1.2% in LE5_[phl+amph]_, whereas amphibole (1.4%) occurs only in LE5_[phl+amph]_ (Table 1).

**Figure 1:**
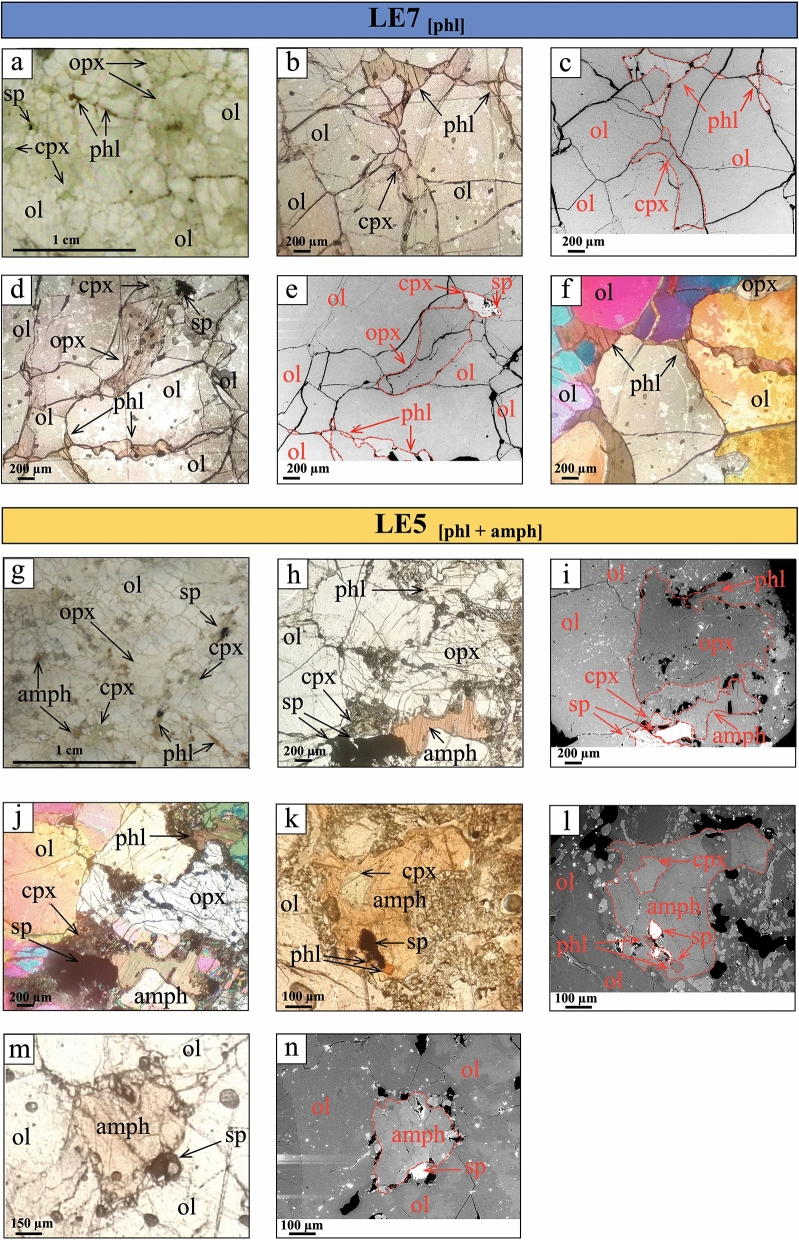
Thin sections images under optical microscope, plane- and cross-polarized transmitted light, and scanning electron microscope in backscattered electron mode (BSE) of Mount Leura peridotite sample LE7_[phl]_ (**a, b, c, d, e, f**) and sample LE5_[phl+amph]_ (**g, h, i, j, k, l, m, n**). (**a**) thin section of LE7_[phl]_ showing the harzburgite mineral assemblage with phlogopite (phl). **b, c, d, e, f** phl micrograins showing textural equilibrium with clinopyroxene (cpx), olivine (ol) and orthopyroxene (opx) in sample LE7_[phl]_. **a, b, d** plane pol. light; **c**, **e** BSE images; **f** cross pol. light. **g** thin section of LE5_[phl+amph]_ showing the harzburgite mineral assemblage with phl and amphibole (amph). **h, i, j, k, l, m, n** microphotographs showing different aspects of the textural relationships between mineral phases, including amphibole and phlogopite. k displays a phlogopite micrograin included in a larger amphibole grain. The latter, with corroded borders, is set in a reaction zone. A cpx micrograin, with corroded edges, is enclosed in the amphibole. **i, l, n** show bright oxides and sulfides included in minerals. **g, h, k, m** plane pol. light; **i, l, n** BSE images; **j** cross pol. light.

Following the Mercier and Nicolas^41^ nomenclature, both samples exhibit protogranular texture with large olivine (2–3 mm in size) characterized by kink-bands, and coarse orthopyroxene (1–2 mm in size) with lobate grain boundaries (Fig. 1). Primary spinel and clinopyroxene are smaller (~ 0.1–0.3 mm in size), and anhedral in shape (Fig. 1). LE7_[phl]_ harzburgite is devoid of small-grained reaction patches and of any clear evidence of host magma infiltrations. In LE7_[phl]_, phlogopite crystals (~ 0.1–0.5 mm in size) occur either as disseminated grains in olivine rich domains, or aligned along former fractures and grain boundaries of olivine and orthopyroxene (Fig. 1a-f). In back-scattered electron (BSE) images, phlogopite show a homogeneous brightness, indicating lack of zoning with respect to major elements (Fig. 1c, e). In LE5_[phl+amph]_, phlogopite occurs as large lobate grains (~ 0.1–0.3 mm in size) in harzburgite matrix (Fig. 1g-j) and as small crystals associated with spinel, clinopyroxene and amphibole (Fig. 1k, l). Amphibole crystals are larger than phlogopite (~ 0.5–1.5 mm in size), anhedral/lobated in shape, like the coexisting peridotite minerals and phlogopite (Fig. 1k-n). In LE5_[phl+amph]_ some amphibole crystals are locally surrounded by fine-grained clinopyroxene, olivine, spinel and glass (Fig. 1k, l).

In both xenoliths, BSE images of phlogopite and amphibole grains, even those close to or embedded in fine-grained reaction zones, show a *quasi*-homogeneous degree of brightness, thus suggesting a modest chemical zoning (Fig. 1l, n).

## Results

### Xenolith bulk composition

LE5_[phl+amph]_ has a higher FeO/MgO ratio and is slightly richer in CaO, Na_2_O, K_2_O and Al_2_O_3_ than LE7_[phl]_, in keeping with the formation of amphibole, in addition to phlogopite (Table 1), and their abundance. The reaction zones did not significantly modify the xenolith’s bulk chemistry. Both samples show large contents of compatible/moderately incompatible minor elements (Ni: 2191–2529 ppm; Cr_2_O_3_: 2051- 2900 ppm; and Mn: 0.11–014 wt% MnO) and are highly depleted of incompatible major element oxides (Al_2_O_3_: 0.21–0.69 wt%; TiO_2_: 0.02–0.06 wt%). Modal and chemical compositions of the two samples agree with harzburgite residues’, as proven by 22–25% of anhydrous, or hydrous (1 wt% H_2_O), melting of fertile lherzolites (PM-DMM-like) at ~ 2 GPa in various experimental studies^42–46^.

### Mineral composition

Major element compositions of peridotite minerals, phlogopite and amphibole are set out in Supplementary Tables 1–6.

The composition of the primary minerals in both samples is homogenous at grain scale, but with an increasing variability at thin-section scale.

Forsterite content [Fo = Mg/(Mg + Fe)_mol_ × 100] in olivine ranges from 89.8 to 91.4 in LE7_[phl]_, and from 89.1 to 93.0 in LE5_[phl+amph]_; the latter larger range may be attributable to a more pervasive late stage metasomatism^47,48^. NiO content lies in the interval 0.33–0.45 wt%.

In both samples, orthopyroxene far from the reaction zones show larger Mg# [Mg/(Mg + Fe) × 100, cations per formula unit; 90.7–93.6] than coexisting olivine. Such a figure agrees with those predicted by the equilibrium partitioning of Mg and Fe between these phases^36^. Al_2_O_3_ and Cr_2_O_3_ vary from 1.08 to 2.90 wt% and from 0.33 to 0.88 wt%, respectively. Ti and Na are negligible. The reported compositional ranges are consistent with a residual character of the rock^46^.

In both samples, clinopyroxene abundance is ≤ 4% (Table 1), with Mg# from 90.4 to 92.9. The TiO_2_ content is lower than 0.40 wt%, whereas Al_2_O_3_ and Cr_2_O_3_ lie in the range of 3.08–4.99 wt% and 1.20–2.40 wt%, respectively. The iron-magnesium partitioning between clinopyroxene and orthopyroxene is close to its equilibrium value^36^ (D = 1.09 ± 0.14).

**Figure 2:**
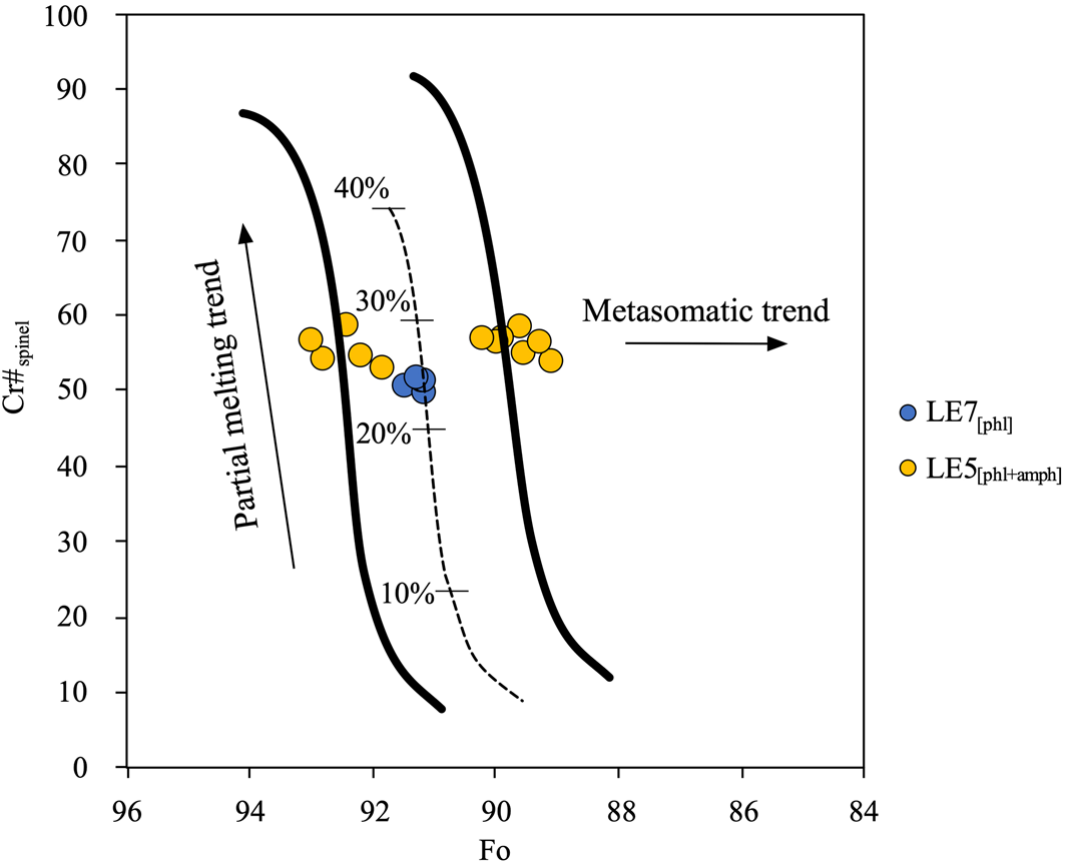
Plot of Cr#_Sp_ [Cr/(Cr + Al)_mol_ × 100] *versus* Fo [Mg/(Mg + Fe)_mol_ × 100] contents in olivine, for Mount Leura (LE) peridotite xenoliths. The olivine-spinel mantle array (OSMA) and melting trend are from Arai^47^. The “metasomatic trend” is generally referred to as the effect of interaction with a migrating fluid/melts.

Spinel have compositions on the (Mg,Fe)Al_2_O_4_-(Mg,Fe)Cr_2_O_4_ join, with Mg > Fe^2+^, Cr > Al and a negligible Ti content. Despite their textural position, the large spinel crystals in LE5_[phl+amph]_ (Fig. 1e) share similar compositions, with Cr#_Sp_ [Cr#_Sp_ = Cr/(Cr + Al) × 100] of 48.3–59.0. In addition, the combination of Cr#_Sp_ with the Fo content of primary olivine points to a high-degree mantle melting (LE7_[phl]_: ~ 22–25%; LE5_[phl+amph]_: ~ 25–28%; Fig. 2), in agreement with the inferences from the bulk rock^42–46^.

The Cr content in spinel, orthopyroxene and clinopyroxene is such that Cr#_Sp_ > Cr#_Cpx_ > Cr#_Opx_ (Supplementary Tables 2–4). This suggests that the compositional evolution of spinel results from a Cr-Al exchange with coexisting pyroxenes during mantle melting^49–51^, thus promoting an increase of the Cr/Al ratio in spinel and the formation of Al-bearing coexisting pyroxene^52^.

Phlogopite, as the only hydrous mineral species (LE7_[phl]_) or coexisting with amphibole (LE5_[phl+amph]_), displays minor oscillations of its major element contents. In particular: Mg# 90.7–92.8; TiO_2_ > 1.5 wt%; Cr_2_O_3_ > 0.8 wt%. Phlogopite in LE5_[phl+amph]_ exhibit slightly higher contents of TiO_2_ and Na_2_O (Supplementary Table 5) than LE7_[phl]_. Phlogopite in LE7_[phl]_ is richer in F (0.119–0.394 wt%), but poorer in Cl (0.020–0.056 wt%) with respect to LE5_[phl+amph]_ (F: 0.110–0.210 wt%; Cl: 0.048–0.073 wt%).

**Figure 3:**
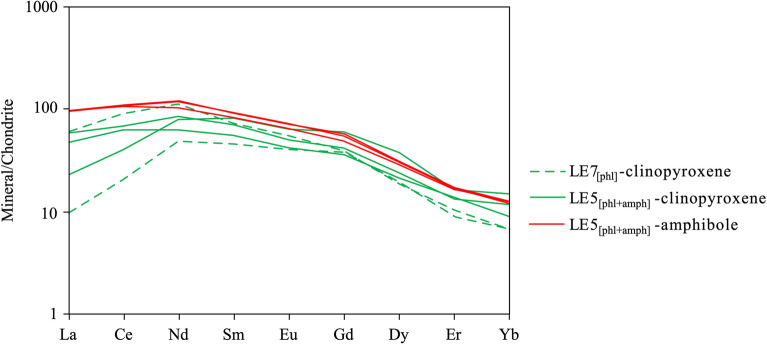
Chondrite normalized^53^ REE patterns of clinopyroxene (green lines) and amphibole (red lines) of Mount Leura xenoliths.

Large amphibole crystals in LE5_[phl+amph]_ (Fig. 1g, i) exhibit (i) Mg# (89.5–90.7), i.e., in the range of the values recorded for the co-existing olivine, orthopyroxene and clinopyroxene, (ii) FeO_tot_, TiO_2_ and Cr_2_O_3_ 3.35–3.82, 1.48–3.12 and 1.50–2.39 wt%, respectively. F and Cl are observed in the intervals 0.100–0.300 and 0.040–0.068 wt%, respectively. Amphibole crystals, without evidences of reaction rims, were extracted for in-situ Mössbauer spectroscopy (SMS) and single crystal diffraction experiments. Many amphibole grains show reaction rims with evidence of late stage trasformation^32,34^; the Mg# values range from 86.9 to 90.1, with large zoning in FeO_tot_ (3.37–4.62 wt%) and TiO_2_ (1.76–3.47 wt%) (Supplementary Table 6). Trace element abundances in clinopyroxene, amphibole and phlogopite, determined by LA-ICP-MS (see “Methods”), are set out in Supplementary Table 7. The chondrite-normalized^53^ Rare Earth Element (REE) signatures of clinopyroxenes in both samples are very similar to each other and to the ones of large amphibole grains of LE5_[amph+phl]_ (Fig. 3). They all exhibit enriched patterns with a weakly positive slope between La_N_ and Nd_N_, followed by a *quasi*-flat trend of Middle Rare Earth Elements (MREE)_N_, and then by a steady decrease of Heavy Rare Earth Elements (HREE)_N_.

Like amphibole, phlogopite in both samples incorporate Nb (and Ta) more efficaciously than the coexisting clinopyroxene. In contrast, REE abundances are very small, and LREE lie below the detection limit (0.1 ppm; Supplementary Table 7). Phlogopite are observed to host also Cs, Rb, Ba, Ni and Cr.

### Fe^3+^/Fe_tot_ determination

Phlogopite and amphibole contain both ferric and ferrous iron, whose proportions are related to the oxygen fugacity conditions^54,55^. The Mössbauer spectroscopy provides measurements of the Fe^3+^ and Fe^2+^ proportions, but there are some limitations due to the amount of sample required. In our case, amphibole and phlogopite were not sufficient for conventional Mössbauer measurements (even using a micro-source), but amphibole crystals exhibited a size suitable for synchrotron Mössbauer spectroscopy (SMS). Unfortunately, the thinness of the phlogopite crystals did not allow reliable measurements even with the latter.

**Figure 4:**
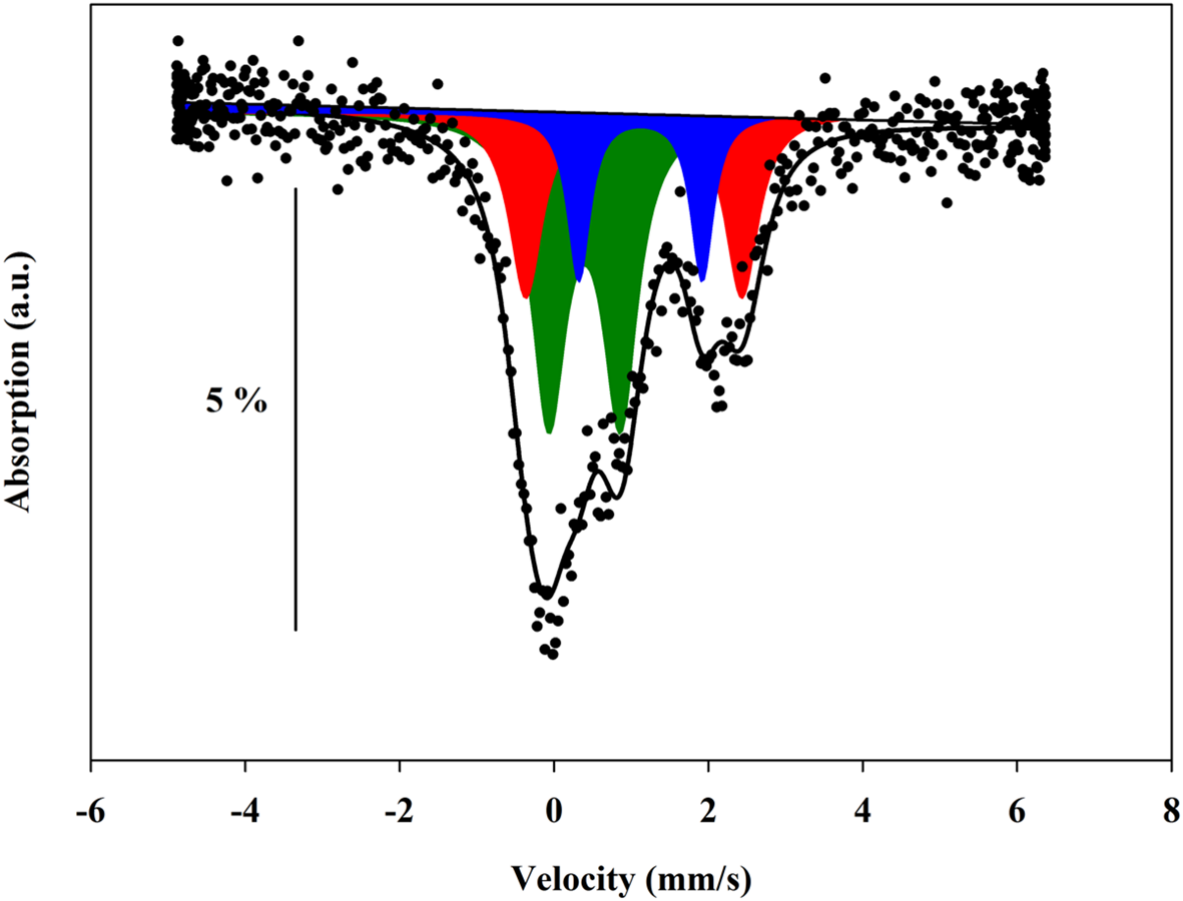
Synchrotron Mössbauer spectrum of the amphibole LE5-AMPH single crystal. Black dots: experimental data; black solid line: full absorption spectrum integral fit; green (Fe^3+^), blue and red (Fe^2+^) areas: individual absorption subspectra. Percentage bar indicates the relative absorption.

Figure 4 shows the SMS absorption spectrum of an amphibole crystal extracted from the LE5_[phl+amph]_ xenolith. The observed Mössbauer absorption pattern is typical of a paramagnetic silicate without impurities and can be satisfactorily modelled by three quadrupole doublets. Two quadrupole doublets (assigned to Fe^2+^) were modelled with an isomer shift (IS) value of 1.1 mm/s, and quadrupole splitting (QS) values of 1.6 mm/s and 2.8 mm/s. The third one, with IS of 0.39 mm/s and QS of 0.93 mm/s, was ascribed to Fe^3+^. Such assignments agree with the vast literature on amphiboles^55–58^. The Fe^3+^ proportion determined from direct measurement of the absorption area is ~ 51% Fe_tot_, which becomes ~ 46% if the corrective strategy of Dyar et al.^56^ is adopted, with an estimated 2σ-uncertainty of about ± 6%.

With reference to the octahedrally coordinated M(1), M(2), M(3) sites and the distorted eight-coordinated M(4) site of the amphibole structure, Fe^3+^ was ascribed to M(2), in agreement with literature^59,60^. The site attribution of Fe^2+^ is more controversial because several different partitioning schemes have been proposed so far. For instance, in the case of the calcic tremolite-actinolite series, Burns and Greaves^55^ ascribed the Fe^2+^ doublets with QS of 1.7–1.9 mm/s and 2.8–2.9 mm/s to the occurrence of Fe^2+^ at M(2) and M(1), respectively. Other authors agreed to assign the doublet with the lowest QS to Fe^2+^ in M(2), but indicated other assignments for the doublet with the highest QS, suggesting an irresolvable combination of Fe^2+^ at [M(1),M(3)] (e.g., Gunter et al.^59^).

As to tremolite, fibrous cummingtonite, grunerite (namely amosite) and anthophyllite, Bancroft et al.^61,62^, Hafner and Ghose^63^, Goldman and Rossman^64^, and Stroink et al.^65^ attributed the Fe^2+^ doublets with QS of 1.5–1.8 mm/s to M(4), and those with QS of 2.6–2.8 mm/s to an irresolvable combination of [M(1),M(2),M(3)].

In our case, the Mössbauer results, complemented by chemical and structural data, confirm that Fe^3+^ is located at the M(2) site and suggest that Fe^2+^ is distributed over M(1) and M(4), with preference for the former (Fig. 4). Accordingly, the Fe^2+^ doublet with QS of 1.6 mm/s (19% Fe_tot_) is assigned to M(4), and Fe^2+^ with QS of 2.8 mm/s (30% Fe_tot_) to M(1).

### Single crystal structure characterization

Both xenoliths contain texturally equilibrated amphibole and/or phlogopite crystals of suitable size for single crystal X-ray diffraction experiments.

Amphibole and phlogopite structures’ details from structure refinements are reported in Supplementary Tables 8–10. The crystal-chemical formulae of the hydrous phases were calculated by combining structure information with the chemical compositions resulting from the average of 8, 5 and 12 wavelength dispersive X-ray spectroscopy analysis points on LE7_[phl]_-phlogopite, LE5_[phl+amph]_-phlogopite and LE5_[phl+amph]_-amphibole specimens, respectively.

In amphibole, hydroxyl groups can be partially replaced by oxygen atoms (Oxo component), thus affecting the distance between the octahedral sites M(1) and M(2). Following Oberti et al.^66^, who correlated the M(1)-M(2) bond length to the degree of OH ↔ O substitution, we measured the M(1)-M(2) distances in the range 3.121–3.127 Å, corresponding to 0.83–0.94 Oxo *per* formula unit (a.p.f.u.), i.e. about 42–47% of O replacing OH *per* site. Taking into account the total content of the iron oxide, the Fe^2+^ and Fe^3+^ proportions determined by SMS, and the Oxo replacement, we propose the following average crystal-chemical formula for the LE5_[phl+amph]_-amphibole:

$$ \begin{aligned} & \left( { \text{Na}_{0.82} \text{K}_{0.17} } \right)_{\Sigma = 0.99} (\text{Na}_{0.11} \text{Ca}_{1.56} )_{\Sigma = 1.77} (\text{Mg}_{3.87} \text{Fe}^{2 + }_{0.22} \text{Fe}^{3 + }_{0.21} \text{Al}_{0.52} \text{Cr}_{0.22} \text{Ti}_{0.26} \text{Mn}_{0.01} \text{Ni}_{0.01} \text{P}_{0.01} )_{\Sigma = 5.33} \\ & \left( {\text{Si}_{6.45} \text{Al}_{1.55} } \right)_{\Sigma = 8} \text{O}_{22} \left[ {\text{O}_{0.83} \left( \text{OH} \right)_{1.16} \text{Cl}_{0.01} } \right]_{\Sigma = 2}  \\ \end{aligned}$$

which, on the basis of the current nomenclature of the amphibole super-group^67^, corresponds to pargasite.

As to phlogopite crystals, we were not able to carry out experimental determinations of the Fe^3+^/Fe_tot_ ratio. We chose to overcome this difficulty using the ratio obtained from amphibole. In our opinion such an approximation is acceptable because:

(i) the structural differences between these two minerals are not expected to have any relevant influence on the Fe oxidation state;

(ii) phlogopite and amphibole, which coexist, are assumed to have formed under the same equilibrium conditions;

(iii) mica crystals from the two xenoliths share very similar compositions.

The results obtained from structure refinements of phlogopite were compared with earlier determinations for mica of similar compositions (see the compilation of Brigatti et al.^68^). This led us to conclude that the phlogopite crystals under investigation do not show any evidence of vacancy occurrence in the octahedrally- and tetrahedrally-coordinated sites.

The crystal-chemical formulae of LE7_[phl]_/LE5_[phl+amph]_-phlogopite crystals were calculated by setting the sum of the octahedrally- and tetrahedrally-coordinated cations equal to 7 and fulfilling electroneutrality by the replacement of OH^-^ with O^2-^. In doing so, both phlogopite samples exhibit a degree of de-hydroxylation of ~ 20% *per* site, corresponding to ~ 0.40 a.p.f.u., as shown by the following crystal-chemical formulae:

LE7_[phl]_-phlogopite:

$$\left( {{\text{K}}_{0.84} {\text{Na}}_{0.14} } \right)_{\Sigma = 0.98} \left( {{\text{Mg}}_{2.44} {\text{Fe}}^{3 + }_{0.10} {\text{Fe}}^{2 + }_{0.10} {\text{Al}}_{0.15} {\text{Cr}}_{0.08} {\text{Ti}}_{0.12} {\text{Ni}}_{0.01} } \right)_{\Sigma = 3} \left( {{\text{Si}}_{2.81} {\text{Al}}_{1.19} } \right)_{\Sigma = 4} {\text{O}}_{10.36} {\text{F}}_{0.05} \left( {{\text{OH}}} \right)_{1.59}$$

LE5_[phl+amph]_-phlogopite:

$$\left( {{\text{K}}_{0.75} {\text{Na}}_{0.19} } \right)_{\Sigma = 0.94} \left( {{\text{Mg}}_{2.38} {\text{Fe}}^{3 + }_{0.11} {\text{Fe}}^{2 + }_{0.11} {\text{Al}}_{0.10} {\text{Cr}}_{0.10} {\text{Ti}}_{0.19} {\text{Ni}}_{0.01} } \right)_{\Sigma = 3} \left( {{\text{Si}}_{2.78} {\text{Al}}_{1.22} } \right)_{\Sigma = 4} {\text{O}}_{10.42} {\text{Cl}}_{0.01} \left( {{\text{OH}}} \right)_{1.57}$$

## Discussion

In many mantle xenolith suites, phlogopite and amphibole occur separately in time and space, and are associated with two distinct types of metasomatism (e.g., silicate versus carbonatite melts: West Eifel, Germany^6^; Ichinomegata, Japan^69^).

On the contrary, the two hydrous phases in Mount Leura’s samples are supposed to have formed simultaneously during the same metasomatic event. They might have achieved equilibrium with the primary anhydrous minerals (i.e., olivine, orthopyroxene and clinopyroxene) prior to the last-stage metasomatic event occurrence, which is likely related to host basalt infiltration that locally perturbed the original chemical and textural features (Supplementary Table 1–6; Table 1; Fig. 1).

### Inter-mineral trace element equilibrium

Once equilibrium is achieved (simultaneous crystallization and sub-solidus re-arrangement), the observable inter-mineral trace element is expected to approximately follow the ideal coefficients (D_i_ = C_i_ mineral1/C_i_ mineral2). Clinopyroxene, which in both xenoliths share the same chondrite normalized REE profile and LREE zoning, and coexisting amphibole yield MREE and HREE partitioning coefficients in agreement with those measured at equilibrium (Fig. 3; ^amphibole/clinopyroxene^ D_REE_: MREE ~ 1.3; HREE ~ 1.1 in silicate ultramafic system^70^). Equilibrium between amphibole and phlogopite (and clinopyroxene) was assessed using elements of different geochemical affinities: Zr-Ti (High Field Strength Elements-HFSE), Sr (Large Lithophile Element-LILE) and F-Cl (halogens). Zr is an element ranging from mildly incompatible to compatible and is preferentially hosted by amphibole rather than clinopyroxene and phlogopite (Table 2). The Zr partitioning coefficient of the phlogopite/clinopyroxene pair from Mount Leura xenoliths is in the narrow range of 0.22–0.51, close to the upper limit experimentally observed, but in agreement with those measured on natural samples (Table 2). The ^amphibole/phlogopite^ D_Zr_ values, obtained for the samples under investigation, approach those from experiments at equilibrium on a mineral/basaltic melt system (Table 2) and overlap those observed in amphibole-phlogopite bearing mantle xenoliths (Table 2).

**Table 2:** Calculated phlogopite/clinopyroxene (^phlogopite/clinopyroxene^D), amphibole/clinopyroxene (^amphibole/clinopyroxene^D), amphibole/phlogopite (^amphibole/phlogopite^D) partition coefficients for Zr, Sr, Ti, F, and Cl of Mount Leura peridotite xenoliths (bolded values), along with values reported in literature where clinopyroxene, phlogopite and amphibole coexist as mineral phases in each experimental run or in natural samples.

	Values	References	Experimental/natural sample
^phlogopite/clinopyroxene^D_Zr_	0.10–0.60	Grégoire et al.^74^	Natural
	0.54	Krmíček et al.^75^	Natural
	0.08–0.16	Adam and Green^70^	Experimental
	**0.22–0.51**	**LE7**_**[phl]**_	
^amphibole/clinopyroxene^D_Zr_	0.91	Grégoire et al.^74^	Natural
	0.92	Adam and Green^70^	Experimental
	**0.63–1.92**	**LE5**_**[phl+amph]**_	
^amphibole/phlogopite^D_Zr_	7.47	LaTourette^76^	Experimental
	3.33–20.00 (4.76–9.09)	Moine et al.^71^ (references therein)	Natural
	11.82	Adam and Green^70^	Experimental
	**2.92–6.23**	**LE5**_**[phl+amph]**_	
^phlogopite/clinopyroxene^D_Sr_	0.50–1.30	Grégoire et al.^74^	Natural
	1.86	Krmíček et al.^75^	Natural
	1.20–1.68	Adam and Green^70^	Experimental
	**0.49–2.24**	**LE7**_**[phl]**_	
^amphibole/clinopyroxene^D_Sr_	2.29	Grégoire et al.^74^	Natural
	1.58	Adam and Green^70^	Experimental
	**2.50–7.61**	**LE5**_**[phl+amph]**_	
^amphibole/phlogopite^D_Sr_	1.87	LaTourette^76^	Experimental
	2.33–3.45 (1.69–3.33)	Moine et al.^71^ (references therein)	Natural
	1.31	Adam and Green^70^	Experimental
	**3.94–4.61**	**LE5**_**[phl+amph]**_	
^phlogopite/clinopyroxene^D_Ti_	3.95–13.10	Grégoire et al.^74^	Natural
	4.16	Krmíček et al.^75^	Natural
	3.08–3.74	Adam and Green^70^	Experimental
	**4.25–8.01**	**LE7**_**[phl]**_	
^amphibole/clinopyroxene^D_Ti_	4.68	Grégoire et al.^74^	Natural
	2.40	Adam and Green^70^	Experimental
	**2.06–8.97**	**LE5**_**[phl+amph]**_	
^amphibole/phlogopite^D_Ti_	0.79–0.89 (0.56–0.68)	Moine et al.^71^ (references therein)	Natural
	0.71	Adam and Green^70^	Experimental
	**0.37–1.22**	**LE5**_**[phl+amph]**_	
^amphibole/phlogopite^D_F_	0.52–2.12	Hauri et al.^77^	Experimental
	0.50–0.53	Adam et al.^72^	Experimental
	0.60–0.66	Flemetakis et al.^73^	Experimental
	**0.06–2.73**	**LE5**_**[phl+amph]**_	
^amphibole/phlogopite^D_Cl_	0.70–0.82	Hauri et al.^77^	Experimental
	0.69–0.70	Adam et al.^72^	Experimental
	0.63–0.86	Flemetakis et al.^73^	Experimental
	**0.42–1.42**	**LE5**_**[phl+amph]**_	

In the case of Ti, ^amphibole/phlogopite^ D_Ti_ is measured in the range 0.46–0.89, in agreement with experimental data by Adam and Green^70^ and Moine et al.^71^ (Table 2). Therefore, even if the coexistence of amphibole and phlogopite in the LE5_[phl+amph]_ sample is texturally affected by last stage metasomatism, the inter-mineral partition coefficients confirm that Zr and Ti attained the equilibrium conditions in the coexisting hydrous minerals and Mount Leura harzburgite matrix (Table 2).

As to Sr, our samples yield ^amphibole/clinopyroxene^ D_Sr_ and ^amphibole/phlogopite^ D_Sr_ larger than earlier experimental determinations, whereas ^phlogopite /clinopyroxene^ D_Sr_ is close to equilibrium measurements (Table 2). This suggests a mobilization and re-distribution of Sr during last stage metasomatism, and the amphibole seem to be more affected than phlogopite.

The halogen concentration in coexisting amphibole and phlogopites is a marker of the nature of the metasomatic melt/fluid that, reacting with peridotite, led to the hydrous phases. Experiments on F and Cl partitioning between amphibole, phlogopite and basanite melts in equilibrium with peridotite^72,73^ show that fluorine is highly incompatible with peridotite minerals, but compatible with amphibole and phlogopite, the latter being the preferred host (^amphibole/phlogopite^ D_F_ ~ 0.5–0.66; Table 2). Chlorine, in turn, is incompatible in peridotite phases but *mildly* incompatible with amphibole and phlogopites (Table 2). In our samples, amphibole core and phlogopite (both core and rim) have F and Cl contents whose partitioning coefficients (observed: ^amphibole/phlogopite^ D_F_: 0.49–0.90; ^amphibole/phlogopite^ D_Cl_: 0.42–0.93) approach those expected at equilibrium (Table 2), whereas amphibole rim seems to record the last metasomatic event, through a remarkable mobility of both F and Cl (observed: ^amphibole/phlogopite^ D_F_ ~ 2.2–2.5; ^amphibole/phlogopite^ D_Cl_ ~ 1.5–3.5). Altogether, the trace element partitioning may be compatible with the following scenery:

(i) phlogopite and amphibole crystals formed through the same metasomatic reaction, and equilibrated with the peridotite mineral assemblage and (ii) the interaction of the xenoliths with the host basalt mainly affected amphibole.

Therefore, the occurrence of phlogopite and phlogopite + amphibole in the harzburgitic mineral assemblage of the Mount Leura xenoliths was also studied to track the geothermal state and redox conditions of the peridotite system, and to formulate the fundamental reactions that may account for such a phase composition.

### Geothermal state and redox conditions

Estimates of oxygen fugacity (*f*O_2_) are important to predict stable phase assemblages in the mantle, in particular OH-bearing phases (amphibole and phlogopite).

The redox conditions of phlogopite + amphibole-bearing peridotite of this mantle domain were assessed through a *f*O_2_ marker calculated from the olivine + orthopyroxene + spinel assemblage, according to the end-member reaction: 6 Fe_2_SiO_4_ (fayalite) + O_2_ = 3 Fe_2_Si_2_O_6_ (orthopyroxene) + 2 Fe_3_O_4_ (spinel), at given *P–T* conditions^37,78,79^.

The equilibrium temperature of this peridotite mineral assemblage was determined using the two-pyroxene geothermometer by Brey and Köhler^36^, and the olivine-spinel geothermometers by Ballhaus et al.^37^ and Jianping et al.^39^. For such an estimate, the equilibrium pressure was set to 1.5 GPa, which lies in the pressure range of the spinel stability. Both samples were equilibrated at similar thermal conditions with an average temperature of ~ 1230 K, though LE7_[phl]_ recorded slightly higher *T*-values (*T*_Opx-Cpx_: 1313 ± 15 K; *T*_Ol-Sp_ 1280 ± 100 K), with respect to LE5_[phl+amph]_ (*T*_Opx-Cpx_: 1175 ± 32 K; *T*_Ol-Sp_ 1151 ± 83 K) (Table 1).

Several studies calibrated the O_2_-fugacity buffered by the olivine-orthopyroxene-spinel mineral equilibrium^39,79,80^ as a function of spinel composition and *P*–*T* conditions. Oxygen fugacity estimates refer to the fayalite-magnetite-quartz (FMQ) buffer (calibration of Frost^40^) and are reported in terms of Δ(FMQ), where Δ(FMQ) = log(*f*O_2_)_rock_–log(*f*O_2_)_FMQ_.

Here, we chose the formulation by Wood^39^ and applied the Wood and Virgo^81^ correction to determine the Fe^3+^/Fe_tot_ ratio in spinel, using data from electron microprobe analyses.

The Wood and Virgo^81^ correction (validated by Davis et al.^54^), substantially improves both accuracy and precision of the Fe^3+^/Fe_tot_ ratio in spinel from EMPA determinations. In particular, such a correction is very effective whenever Fe^3+^/Fe_tot_ is required at the grain scale, and provided that secondary oxidation did not occur during the spinel thermal history subsequent to its crystallization. In fact, if spinel crystals undergo oxidation (i.e., increasing environmental oxygen fugacity), they become non- stoichiometric^82,83^. In our case, no evidence of oxidation was observed, neither in spinel nor in other coexisting phases, and the Δ(FMQ) values from the spinel composition, using EMPA data and adopting the Wood and Virgo^81^ correction, coherently point to a homogeneous and reducing environment.

Uncertainties in calculated *f*O_2_ were estimated as ~ 0.4 log units, at *f*O_2_ lower than about FMQ-1 (see Davis et al.^54^). Notably, LE7_[phl]_ and LE5_[phl+amph]_ harzburgites recorded almost identical fugacity conditions [–1.83 ± 0.36 and –1.97 ± 0.35, in terms of Δ(FMQ); Table 1], which reflects similar and reducing conditions, well in the graphite stability field if compared with typical mantle spinel peridotite^83^. As a consequence, we conclude that LE7_[phl]_ and LE5_[phl+amph]_ shared the same mantle conditions, further reflected by the chemical similarity of the mica crystals in both samples (Supplementary Tables 5–6).

### Phlogopite-amphibole stability model

The stability of phlogopite and phlogopite + amphibole in the peridotite system is governed by the key-equation of equilibrium, i.e.:

1 $${\sum }_{k}{\nu }_{k}{G}_{k}={\sum }_{j}{\nu }_{j}{G}_{j}$$

where ν and G are stoichiometric coefficients and molar Gibbs energy of the involved phases, respectively. Under the constraints of (1), the occurrence of pargasite, phlogopite, spinel, clinopyroxene and olivine in the LE5_[phl+amph]_ harzburgite was modelled by the following approximate equation:

2 $${\text{pargasite}} + {\text{forsterite}} = {\text{aspidolite}} + 2{\text{ diopside}} + {\text{spinel}},$$

where we simplified the mineral phases by means of their end members:

pargasite [NaCa_2_(Mg_4_Al) (Si_6_Al_2_)O_22_(OH)_2_], forsterite [Mg_2_SiO_4_], spinel [MgAl_2_O_4_], diopside [CaMgSi_2_O_6_] and aspidolite [NaMg_3_(AlSi_3_)O_10_(OH)_2_].

This allowed us to estimate the *P*–*T* equilibrium-like conditions of LE5_[phl+amph]_ (hereafter: equilibrium conditions *tout court*). The calculations related to (2) were carried out under the constraint provided by Eq. (1), splitting the Gibbs energy into two contributions: an integration along an isotherm and an integration along an isobar, as discussed by Curetti et al.^84^. We chose to develop our calculations following two general principles: (i) using as few phases as possible to model Eq. (2), in order to limit the uncertainty due to the experimental error on the observables involved in the calculations; (ii) selecting literature data, for which the experimental uncertainties are comparatively small. Calorimetric and thermo-elastic data of the involved mineral phases are set out in Supplementary Table 11. In particular, thermo-elastic data were chosen paying special care to their uncertainties, to minimize the error that propagates on the energy deformation calculated along the related isobar (Supplementary Table 11). Δ*G*, i.e., the Gibbs energy difference between the left-hand side member and right-hand side member of (2), was split into two parts: (i) Δ*G*_s-t_, which accounts for all contributions (static and thermal components) except for configuration entropy; (ii) Δ*G*_conf_, which expresses the configuration entropy contribution. Preliminary tests were performed using 5 × 5 × 5 supercells, semi-empirical potentials and lattice dynamics via the GULP code^85^ to model the effects induced by cation order–disorder in the minerals under investigation. We estimated that Δ*G*_s-t_ changes comparatively little (~ 5–7%) with respect to the end-members, if the mineral compositions observed in the present investigation are used. Δ*G*_conf_, in turn, affects Δ*G* by 35–50%, in the range from 800 to 1400 K. For this reason, we chose to determine Δ*G*_s-t_ from end-members, and Δ*G*_conf_ from the crystal-chemical results obtained by structure refinements and Mössbauer spectroscopy. For olivine, clinopyroxene and spinel we used the chemical compositions (Supplementary Tables 1, 3–4) from EMPA, and assumed in each crystal structure the cation partitioning that maximizes configuration entropy.

**Figure 5:**
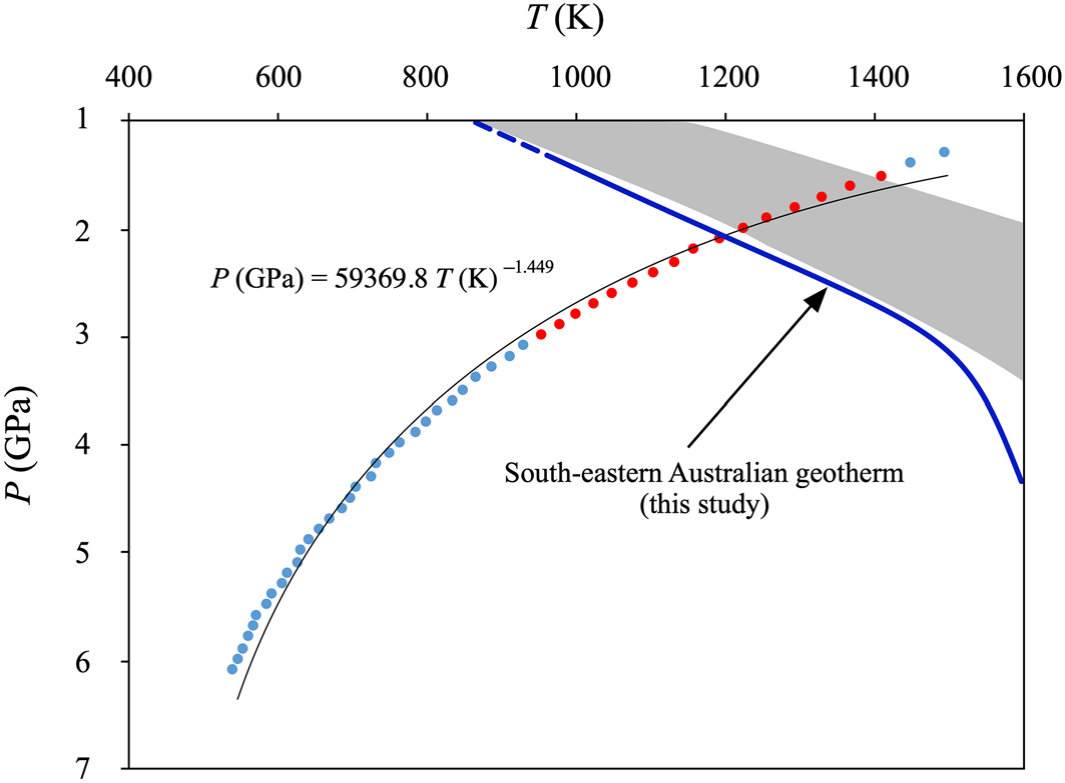
The dot line shows physically sound *P*–*T locus* fulfilling the reaction pargasite + forsterite = Na-phlogopite + 2 diopside + spinel. Marked in blue and in red are the *P–T* regions geologically incoherent and coherent with (spinel-bearing) mantle lithologies, respectively. The modelled *P–T locus* crosses at ~ 2.3 GPa/1190 K the geotherm calculated for this study (blue thick line) from the analysis of surface heat flow data and combining seismic tomography gravity data^88,89^. This *P–T* path is slightly “cooler” with respect to the Australian geotherm’s field (grey field) from literature^86,87^.

The physically sound *P*–*T locus* fulfilling Eqs. (1–2) stretches over the interval 1.3–6.1 GPa/540–1500 K (Fig. 5). The resulting equilibrium *P–T* curve can be approximated by the following equation:

*T*(K) = −4.6966 *P*^3^ + 86.63 *P*^2^ − 619.83 *P* + 2160.5 (*P* in GPa), which yields a negative Clapeyron slope, (∂*P*/∂*T*)_equilibrium_. The presence of Cr-Al spinel in the xenoliths, in combination with detailed geological and petrological studies of the xenolith-bearing volcanic region^27,32,34^, allowed us to restrict the pressure interval to the more realistic range of 1.3–3.0 GPa. In this interval the negative Clapeyron slope of the *P–T* curve has an average value in the range 1.3–3.0 GPa of –0.003 GPa K^–1^. The modelled *P–T locus* crosses the Australian geotherms’ field, calculated by heat-flow measurements and xenolith samples^86,87^, in the lower end at ~ 2.2 GPa /1210 K and in the upper end at ~ 1.4 GPa /1400 K (Fig. 5). The Australian mantle domain that stabilized the Mount Leura xenolith mineral assemblages under study is placed in this region. Using the recent thermal model of the crust and upper mantle of the Australian continent, obtained from the analysis of surface heat flow data and combining seismic tomography and gravity data^88,89^, it was possible to calculate the geotherm of this mantle column down to 200 km of depth in the proximity of the xenolith location (38°14′41.0"S; 143°09′27.5"E). The new proposed geotherm (Fig. 5) takes into account the mantle xenolith’s petrological evidence (part of this study) of the extensive re-fertilization that occurred during the Proterozoic tectonic events^88,89^. The resulting geotherm, specifically related to the mantle column of the Mount Leura xenoliths, is slightly “cooler” than the Australian mantle geotherms’ field (2.3 GPa /1290 K) and intersects the modelled *P*–*T locus* of the amphibole-phlogopite equilibrium at ~ 2.3 GPa/1190 K. This *P*–*T* point represents the plausible thermal-baric conditions of the mantle region (~ 70 km depth) related to the Mount Leura xenolith provenance.

On the basis of these data, the south-eastern Australian geotherm shows a sub-linear trend up to 4 GPa (~ 150 km depth), in agreement with the previous thermal models, and then it becomes convection-driven, well before the previous estimates (Fig. 5). This probably implies that beneath the Newer Volcanic Province there is an increasing efficiency of thermal perturbations in the deeper mantle region.

### Metasomatic fluid stability conditions

We used the equilibrium *P*–*T* curve to constrain the geothermobarometric conditions at which circulating fluids/volatile rich melts led to hydrous phases. Phase and chemical compositions along with textural observations suggest the following equation:

3 $$\begin{aligned} & 2\left( {{\text{K}}_{0.85} {\text{Na}}_{0.15} } \right){\text{Mg}}_{3} \left( {{\text{AlSi}}_{3} {\text{O}}_{10} } \right)\left( {{\text{OH}}} \right)_{{2\;{\text{phlogopite}}}} + {\text{Mg}}_{2} {\text{SiO}}_{{4\;{\text{forsterite}}}} = 7/2{\text{Mg}}_{2} {\text{Si}}_{2} {\text{O}}_{{6\;{\text{enstatite}}}} \\ & \quad + {\text{MgAl}}_{2} {\text{O}}_{{4\;{\text{spinel}}}} + 2\left( {{\text{H}}_{2} {\text{O}} + 1/2\left( {{\text{K}}_{0.85} {\text{Na}}_{0.15} } \right)_{2} O} \right)_{{{\text{Fluid}}}} . \\ \end{aligned}$$

The fluid composition is designed to guarantee a mass balance between the left- and right-hand side members of the equation above.

Let us consider a *virtual reference fluid* (VRF; *G*_Fluid,0_) of composition H_2_O + 1/2 (K_0.85_Na_0.15_)_2_O, which represents an ideal fluid-like system (see “Experimental Methods and modelling”), whose *G*_Fluid,0_ (i.e. molar Gibbs energy) is *formally* modelled as follows:

4 $$G_{{\text{Fluid,0}}} = E_{{{\text{H2O}}}} + 1/2\{ xE_{{{\text{K2O}}}} + (1 - x)E_{{{\text{Na2O}}}} \} - T \times S_{{\text{Fluid,Mixing}}} + \{ H(T) - T \times S\}_{{\text{Fluid,Thermal}}} + G_{{\text{Fluid,Exc}}} .$$

*E*_H2O_, *E*_K2O_ and *E*_Na2O_ are the quantum energies of H_2_O, K_2_O and Na_2_O (crystals);

*S*_Fluid,Mixing_ is the mixing entropy of an ideal gas, composed of K, Na, H and O *independent* atoms;

{*H*(*T*)-*T* × *S*}_Fluid,Thermal_ is calculated by a *C*_*P*_ set equal to 3 k per atom, for *T* ≥ 273 K. At lower *T*, *C*_*P*_ is taken linear, with *C*_*P*_(*T* = 0 K) = 0;

*G*_Fluid,Exc_ is modelled using the activity coefficients for K^+^, Na^+^ and OH^-^ in aqueous solution.

We would like to underline that it is not our aim to develop any physically consistent VRF, but to set up a fictitiously “*over-stable*” fluid-like virtual system.

Equation (3) can be expressed in a general form assuming that (i) all members undergo forward–backward transformations and behave as if they were components, and (ii) the Gibbs energy of each phase is treated like the composition independent part of the chemical potential of a component (γs are set to unity, initially). The resulting chemical equilibrium constant, *K*_*eq*_, is written as

$$\ln \left( {K_{eq} } \right) = - \frac{{\Delta G_{j}^{k} }}{RT} = \ln \left( {\frac{{\mathop \prod \nolimits_{k} x_{k}^{{\nu_{k} }} }}{{\mathop \prod \nolimits_{j} x_{j}^{{\nu_{j} }} }}} \right),$$

where $\Delta G_{j}^{k}$ is the Gibbs energy difference in (3) between left-hand side member and right-hand side member, the latter containing VRF formulated via Eq. (4); *K*_*eq*_ coincides with the concentration quotient, in the present case. Equation (3) virtually shifts leftwards, if *K*_*eq*_ > 1, and rightwards, if *K*_*eq*_ < 1. We chose to describe a deviation of *K*_*eq*_ from 1 by introducing a fictitious activity coefficient that is arbitrarily attributed to the fluid-component, namely 2*RT ln*(*γ*_Fluid_) = $\Delta {G}_{j}^{k}$. Eventually, for bare convenience of notation, we set *γ*_Fluid_ = *f*_Fluid_/*f*_Fluid,0_, and thence Δ*log*(*f*_Fluid_) = *log*(*f*_Fluid_)–*log*(*f*_Fluid,0_). Note that, on an alternative viewpoint, $\Delta {G}_{j}^{k}$ can be seen as the “additional” excess Gibbs energy required for VRF to ideally achieve equilibrium according to Eq. (3). Altogether, Δ*log*(*f*_Fluid_) <  < 0 implies that Eq. (3) is shifted leftwards, namely a very small amount of “*over-stable*” VRF is expected.

Calculations were performed by a combination of:

(i) quantum-mechanics (CRYSTAL code^90^), for static energy contributions;

(ii) semi-empirical potentials, for phonons related energy terms via lattice dynamics (GULP code^85^), such as zero-point energy and calorimetric contributions below room temperature;

(iii) thermo-elastic data are set out in Supplementary Table 11.

We chose to follow such an approach to guarantee the highest comparability between Gibbs energy values, which play an important role in the determination of Δ*log*(*f*_Fluid_).

**Figure 6:**
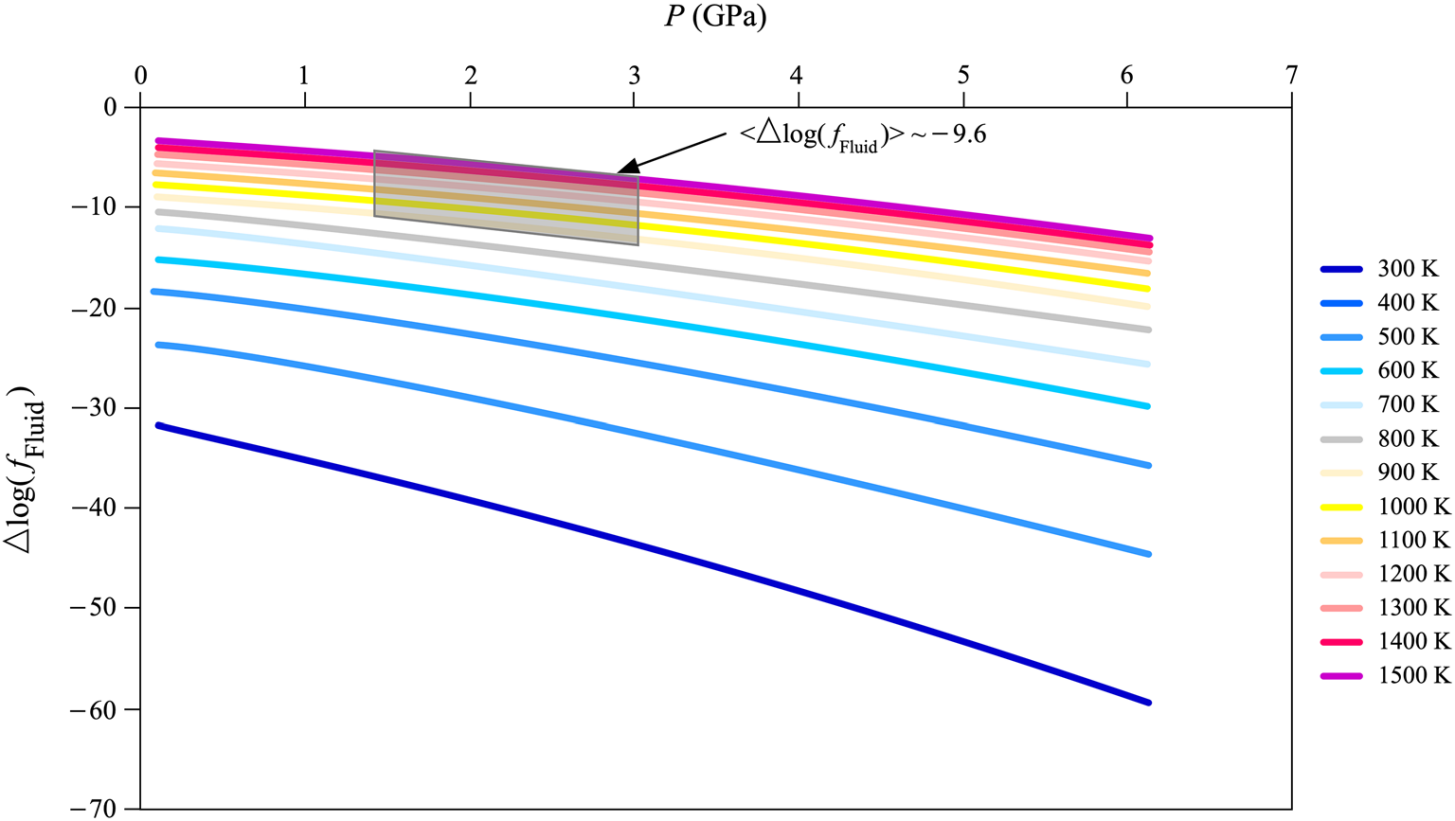
Δ*log*(*f*_Fluid_) = *log*(a_Fluid_) = *log*(*f*_Fluid_)–*log*(*f*_Fluid,0_) of the reaction 2 (K,Na)-phlogopite + forsterite = 7/2 enstatite + spinel + 2 fluid(Na_2_O-K_2_O,H_2_O) along isotherms (300–1500 K) as a function of pressure (*P*). In the *P–T* range of petrological interest (i.e., 900–1500 K and 1.5–3.0 GPa; grey bar), the mean value of Δ*log*(*f*_Fluid_) (< Δ*log*(*f*_Fluid_) >) is ~ −9.6. This means that a large amount of energy should be provided to the system to maintain the fluid phase coexisting with phlogopite (and amphibole).

The Δ*log*(*f*_Fluid_) as a function of pressure and along isotherms (300–1500 K) is reported in Fig. 6. Notably, over the *P*–*T* range of petrological interest, i.e., 900–1500 K and 1.5–3.0 GPa, the mean value of Δ*log*(*f*_Fluid_) (< Δ*log*(*f*_Fluid_) >) is ~ –9.6. This large negative value shows, as stated above, that a hypothetical metasomatic fluid tends to occur in a very small amount, if at all. Therefore, the coexistence of fluid at equilibrium with phlogopite is unlikely, in keeping with petrographic observations, which do not show any evidence of residual fluids in association with phlogopite crystals.

It follows that Na–K aqueous fluids (or Na–K hydrous melt) cannot flow over long distances throughout the mantle as they are highly reactive with the ultramafic system.

All this is in keeping with the observations of Huang et al.^91^, who claim an increase of wettability of H_2_O because of the addition of NaCl, in an ultramafic environment. In our case, owing to the absence of saline brine in the fluid inclusions of the mineral samples, we conclude that the K-Na aqueous fluids, originated in the same “harzburgitic system” as the Mount Leura mantle fragments and immediately promoted the formation of phlogopite and amphibole, and these minerals achieved equilibrium with the ultramafic system.

According to the comparatively low *f*O_2_ values [–1.83 ÷ –1.97 Δ(FMQ)] of LE7_[phl]_ and LE5_[phl+amph]_ harzburgites, such an environment is more reducing than it would be expected from the occurrence of phlogopite and amphibole in spinel bearing peridotites. In fact, this mineral assemblage is commonly associated with an average Δ(FMQ) of ~  ± 1, and several studies ascribe the role of O_2_ buffers to hydrous phases^6,92,93^.

The relatively reducing conditions of LE7_[phl]_ and LE5_[phl+amph]_ harzburgites may indicate (i) that peridotite lacks a sufficient amount of phases (amphibole/phlogopite, but also spinel) capable of buffering O_2_ (which is not our case, as phlogopite and amphibole are estimated to be about 2.5 wt%, in total), and/or (ii) that full molecular dissociation of the fluid takes place, followed by incorporation of the O-H-K-Na species into (OH)-K-Na-bearing mineral phases (phlogopite and amphibole). The modelled metasomatic fluid, having Δ*log*(*f*_Fluid_) <  < 0 points to this second situation, thus leading to a peridotite metasomatized ambient, characterized by reducing conditions^58,92,93^.

It is worth noting that near Mount Leura there are Bullen-Merri and Gnotuk maars, two volcanic crater-lakes probably formed by diatremes, which host spinel lherzolites with coexisting amphibole and phlogopite, containing F and Cl^31,94^. Waters of Lake Bullen-Merri are “brackish” and those of Lake Gnotuk are even hyper-saline (twice as salty as seawater). Among the various hypotheses about the origin of hyper-saline waters of the hydrologically closed maar crater lakes^95^, we venture upon that of a mantle source with amphibole and phlogopite (F and Cl bearing) for the local magmatism. On the basis of the experimental results, 5% partial melting of a metasomatized mantle sources (amph:phl = 2:4), produce primary magmas with fluorine contents of ~ 8500 ppm (using ^ampbibole-phlogopite/melt^ D_F_ < 1) and F/Cl between 5 and 10 ^73^. All this suggests that magmas may have reached the surface with their halogen budgets, which could be preserved and concentrated in the subsequent shallow geological structures^95^.

In conclusion, a novel perspective is applied to the mineral assemblage of olivine + clinopyroxene + orthopyroxene + spinel + phlogopite + amphibole in mantle xenoliths and it can provide further restraints about the effective reducing/oxidizing state of the lithospheric mantle. In addition, this petrological study provides a new finding for a potential geotherm in the southeast Australian upper mantle, which is fully consistent with the results of previous thermal models^86–89^.

### Experimental methods and modelling

Bulk rock major element compositions were determined by wavelength dispersive X-ray fluorescence spectroscopy (WDXRF) on pressed powder pellets at the Department of Physics and Earth Sciences of the University of Ferrara (Italy), using an ARL Advant-XP spectrometer, following the full matrix correction method proposed by Lachance and Traill^96^. Accuracy is generally lower than 2% for major oxides and 5% for trace elements; the detection limit for the latter ranges from 1 to 2 ppm. Volatile contents were determined by loss on ignition (LOI) at 1000 °C.

Polished petrographic thin sections were prepared for optical microscopy observations and in-*situ* chemical analyses. The statistical significance of the petrographic and crystal-chemical analyses was guaranteed by cutting two different slices of the same xenolith, so that two thin sections *per* sample were obtained and analyzed.

Backscattered electron (BSE) images were recorded by a ZEISS EVO MA 15 scanning electron microscope (SEM) at the Department of Physics and Earth Sciences of the University of Ferrara (Italy). This instrument is equipped with an SDD detector, and employs a LaB6 filament as an electron source. The thin sections were studied at 20 kV and 8.5 mm working distance, at high vacuum conditions. Major element compositions of cores and rims of the primary minerals were determined using a JEOL JXA-8900 wavelength dispersive X-ray spectroscopy (WDS) electron microprobe, at the Earth Sciences Department of the University of Milan (Italy). The system was operated with an accelerating voltage of 15 kV, a beam current of 5 nA, a counting time of 30 s on the peaks and 10 s on the backgrounds, and a beam spot size of 10 µm, the latter to avoid sample sublimation. The following standards were used: graftonite for P, Fe, and Mn; grossular for Si, Al, and Ca; K-feldspar for K; forsterite for Mg; niccolite for Ni; and omphacite for Na. The halogen contents in amphibole and phlogopite were measured using natural topaz as reference material and following the analytical protocol of Zhang et al.^97^. The EMPA detection limits for F and Cl are ~ 0.07 and ~ 0.015 wt%, respectively.

Trace element analyses of clinopyroxene, amphibole and mica were performed by LA-ICP-MS, at the Istituto di Geoscienze e Georisorse, C.N.R., Pavia (Italy), using PerkinElmer SCIEX ELAN DRC-e quadrupole mass spectrometer. Helium was used as carrier gas and mixed with Ar downstream of the ablation cell. NIST SRM 610 was used as external standard, while Ca was the internal standard for clinopyroxene and amphibole and Si was the internal standard for phlogopite. Data reduction was performed using the Glitter software. Precision and accuracy were assessed from repeated analyses of the BCR-2 g reference material and usually resulted in being better than 10%. The laser was operated at a repetition rate of 10 Hz, with a pulse energy of ~ 35 mJ. Spot diameter was typically 40–50 μm. Single crystals of phlogopite and amphibole were extracted for X-ray diffraction experiments (XSC) and in-*situ* synchrotron Mössbauer spectroscopy (SMS).

XSC experiments were carried out on single crystals of mica (LE7-MICA 1/8/15) from LE7_[phl]_, and of amphibole (LE5-AMPH 2/3/4/5) and mica (LE5-MICA 1/3/5) from LE5_[phl+amph]_. The diffraction intensities of the crystals were collected using the Gemini R Ultra X-ray diffractometer at the CrisDi Interdepartmental Centre of the University of Torino (Italy), equipped with a Ruby CCD detector, using a monochromatic MoKα radiation and X-ray tube operating at 50 kV and 40 mA. The 171.37.35 version of CrysAlysPro software (Agilent Technologies) was used for data reduction (integration of the intensity spots, absorption and Lorentz-polarization corrections). Structure refinements were performed by the codes Schelx-TL and Jana2006, starting from the atomic coordinates reported in literature for phlogopite^68^ and pargasite^98^. The atomic positions were refined without restraints, save the symmetry constraints; electron occupancies and anisotropic thermal factors of the crystallographic sites were refined using the ionic scattering factors of the more abundant atomic species, in particular: Si^4+^ for T-site, Mg^2+^ for M(1)-M(2)-M(3)-sites, Ca^2+^ for M(4)-site, and Na^+^ for A-site. The “large” A-site of amphibole was filled by cations with a partially disordered arrangement, characterized by three different positions^99^.

Energy-domain in*-situ* SMS measurements were performed on amphibole single crystals, LE5-AMPH, at the Nuclear Resonance beamline ID18^100^ of the European Synchrotron Radiation Facility (ESRF), Grenoble (France), in multi-bunch (7/8 + 1) mode. The SMS instrument is equipped with a nuclear resonant monochromator and employs pure nuclear reflections of an iron borate (^57^FeBO_3_) single crystal^101^. The source provides ^57^Fe resonant radiation at 14.4 keV within a bandwidth of 6 μeV, which is tuneable over an energy range of ± 0.6 μeV^101^. The X-rays beam emitted by the SMS was focused onto a 16-vertical × 15-horizontal μm^2^ spot size, at the full width half maximum. Before and after each sample measurement, the SMS linewidth was determined using a K_2_Mg^57^Fe(CN)_6_ reference single line absorber. The velocity scale (± 5 mm/s) was calibrated by a 25 μm-thick natural α-Fe foil. The small cross section, high brilliance and the fully resonant and polarized nature of the beam allowed rapid data collections (approximately 2 h).

The spectra were fitted with a full transmission integral and pseudo-Voigt line shape using the software package MossA^102^. The single line spectra were modelled by a normalized Lorentzian-squared source line shape. A linear function was used for background.

Equation (4) was developed considering a *virtual* fluid system of composition H_2_O + 1/2 (K_0.85_Na_0.15_)_2_O, with a Gibbs energy lower than that expected for a physical fluid sharing the same composition. We start from (i) solid oxides K_2_O, Na_2_O and H_2_O, having at 0 K a total energy *E*_0_-Δ*E*, where *E*_0_ and -Δ*E* (Δ*E* > 0) are the free atoms’ energy and crystal formation energy, respectively. Such a system is (ii) heated up to *T*_0_ = 273 K, assuming a specific heat *per atom* at constant pressure given by

$$C_{P} = \left( {\frac{T}{{T_{0} }}} \right)3 k,$$

that is an overestimate of *C*_*P*_ for a solid mixture with the phase composition of H_2_O + 0.425 K_2_O + 0.075 Na_2_O ^103,104^.

At 273 K, (iii) the atoms are split apart by an external work equal to Δ*E*. This leads to a mixture of non-interacting monoatomic gases, with *P* → ε, *T*_1_ > *T*_0_ in keeping with the energy equipartition theorem, and *V* ∝ 1/ε, following the gas EoS. We neglect *T*_1_, and start considering the gas mixture from *T*_0_, for the reason below. For *T* > *T*_0_ (iv) we set *C*_*P*_ as large as 3 k per atom, which is an overestimate for an ideal monoatomic gas. The developed model relies upon a general overestimation of *C*_*P*_. Given that

$$H - TS = \int_{0}^{T} {\left( {1 - \frac{T}{{T^{\prime}}}} \right)} C\left( {T^{\prime}} \right)_{P} dT^{\prime} > \int_{0}^{T} {\left( {1 - \frac{T}{{T^{\prime}}}} \right)} C\left( {T^{\prime}} \right)_{P}^{overestimated} dT^{\prime}$$

then the use of an overestimated *C*_*P*_ yields an underestimated thermal contribution to the Gibbs energy, thus fictitiously boosting stability of the related system. Mixing entropy due to the gas mixture is taken into account (*S*_Fluid,Mixing_). We then proceed to compress (v) the monoatomic gas mixture, at *T*. The isothermal compression of the system, changes its Gibbs energy in terms of

$$\Delta G = \int_{{P_{1} }}^{{P_{2} }} {VdP} ,$$

whose positive contribution (*P*_2_ > *P*_1_) is neglected here. Possible condensation processes upon compression cannot lead to an exothermic energy exchange larger than -Δ*E*. Eventually, we complete this modelling of a virtual “*over*-*stable*” fluid introducing an additional, though uninfluential, excess contribution (*G*_Fluid,Exc_) from an aqueous solution using the following equilibrium chemical equations:

$${\text{K}}_{2} {\text{O}} + {\text{H}}_{2} {\text{O}} = 2{\text{K}}({\text{OH}}) = 2{\text{K}}^{ + } + 2({\text{OH}})^{ - }$$

$${\text{Na}}_{2} {\text{O}} + {\text{H}}_{2} {\text{O}} = 2{\text{Na(OH)}} = 2{\text{Na}}^{ + } + 2({\text{OH}})^{ - } ,$$

according to Ferse^105^ and taking the lowest activity coefficients reported there.

(K,Na)-phlogopite, in turn, was modelled as a linear combination of the phlogopite and aspidolite Gibbs energy values. The excess Gibbs energy of the isomorphic replacement involving K and Na in phlogopite, given the composition of (3), was estimated to be ~ 0.01–0.03 kJ/mol in terms of lattice contribution^106^ and therefore neglected.

Calculations were carried out using both quantum mechanics and semi-empirical modelling. Quantum mechanics was employed to calculate the lattice energy of the involved phases, adding the vibrational contributions determined by semi-empirical potentials and lattice dynamics in quasi-harmonic approximation. In the case of lattice dynamics modelling (GULP code^85^), semi-empirical potentials were taken from Catlow^107^, available from the repository www.ucl.ac.uk/klmc/Potentials/Library/catlow.lib. Quantum mechanics calculations rely upon an *ab*-*initio* Hartree–Fock (16%)-DFT (84%) hybrid approach (CRYSTAL code^90^), adopting a Hamiltonian based on the SOGGA (Second Order GGA) functional in combination with the PBE correlation functional^108^. Computational details are reported in Merli and Pavese^109^. Gaussian basis set (name as recorded in the repository of the CRYSTAL site www.crystal.unito.it/Basis_Sets) are: H_pob_TZVP_2012 for H, O_8-411d1_bredow_2006 for O, Na_8-511(1d)G_baranek_2013_NaNbO3 for Na, Mg_8-511d1G_valenzano_2006 for Mg, Al_85-11G*_catti_1994 for Al, Si_88-31G*_nada_1996 for Si, K_pob_TZVP_2012 for K.

## Supplementary Information

Supplementary Information.

## References

[1] Mitchell RH (1986). Kimberlites: Mineralogy, Geochemistry and Petrology.

[2] Tappe S, Foley SF, Jenner GA, Kjarsgaard BA (2005). Integrating ultramafic lamprophyres into the IUGS classification of igneous rocks: rational and implications. J. Petrol..

[3] Reguir EP, Chakhmouradian AR, Halden NM, Malkovets VG, Yang P (2009). Major- and trace-element compositional variation of phlogopite from kimberlites and carbonatites as a petrogenetic indicator. Lithos.

[4] Kargin AV, Sazonova LV, Nosova AA, Lebedeva NM, Kostitsyn YuA, Kovalchuk EV, Tretyachenko VV, Tikhomirova YS (2017). Phlogopite in mantle xenoliths and kimberlite from the Grib pipe, Arkhangelsk province, Russia: evidence for multi-stage mantle metasomatism and origin of phlogopite in kimberlite. Geosci. Front..

[5] Canil D, Scarfe CM (1989). Origin of phlogopite in mantle xenoliths from Kostal Lake, Wells Gray Park, British Columbia. J. Petrol..

[6] Shaw CSJ, Lebert BS, Woodland AB (2018). Thermodynamic modelling of mantle-melt interaction evidenced by veined wehrlite xenoliths from the Rockeskyllerkopf Volcanic Complex, West Eifel Volcanic Filed, Germany. J. Petrol..

[7] Zanetti A, Mazzucchelli M, Rivalenti G, Vannucci R (1999). The Finero phlogopite peridotite massif: An example of subduction related metasomatism. Contrib. Mineral. Petrol..

[8] Zinngrebe E, Foley SF (1995). Metasomatism in mantle xenoliths from Gees, West Eifel, Germany: evidence for the genesis of calc-alkaline glasses and metasomatic Ca-enrichment. Contrib. Mineral Petrol.

[9] Ionov DA, Ashchepkov I, Jagoutz E (2005). The provenance of fertile off-craton lithospheric mantle: Sr-Nd isotope and chemical composition of garnet and spinel peridotite xenoliths from Vitim, Siberia. Chem. Geol..

[10] Witt-Eickschen G, Seck HA, Mezger K, Eggins SM, Altherr R (2003). Lithospheric mantle evolution beneath the Eifel (Germany): Constraints from Sr-Nd-Pb isotopes and trace element abundances in spinel peridotite and pyroxenite xenoliths. J. Petrol..

[11] Kaczmarek MA, Bodinier JL, Bosch D, Tommasi A, Dautria JM, Kechid SA (2016). Metasomatized mantle xenoliths as a record of the lithospheric mantle evolution of the Northern Edge of the Ahaggar Swell, in Teria (Algeria). J. Petrol..

[12] Harte, B. Mantle peridotites and processes-The kimberlite sample in *Continental basalts and mantle xenoliths* (eds. Hawkesworth, C.J. & Norry, M.J.) 46–91 (Shiva: Cheshire, UK, 1983).

[13] Coltorti M, Bonadiman C, Faccini B, Gregoire M, O’Reilly SY, Powell W (2007). Amphiboles from suprasubduction and intraplate lithospheric mantle. Lithos.

[14] Mandler BE, Grove TL (2016). Controls on the stability and composition of amphibole in the Earth’s mantle. Contrib. Mineral. Petrol..

[15] Safonov O, Butvina V, Limanov E (2019). Phlogopite-forming reactions as indicators of metasomatism in the lithospheric mantle. Minerals.

[16] Dawson JB, Smith JV (1977). The MARID (mica-amphibole-rutile-ilmenite-diopside) suite of xenoliths in kimberlite. Geochim. Cosmochim. Acta.

[17] Erlank, A. J., Waters, F. G., Hawkesworth, C. J., Haggerty, S. E., Allsopp, H. L., Rickard, R. S. & Menzies M. Evidence for mantle metasomatism in peridotite nodules from the Kimberley pipes, South Africa in *Mantle Metasomatism* (eds. Menzies, M.A. & Hawkesworth, C.J.) 221–311 (Academic Press, London, 1987).

[18] van Achterbergh E, Griffin WL, Stiefenhofer J (2001). Metasomatism in mantle xenoliths from the Letlhakane kimberlites: Estimation of element fluxes. Contrib. Mineral. Petrol..

[19] Grégoire M, Bell DR, Le Roex AP (2002). Trace element geochemistry of phlogopite-rich mafic mantle xenoliths: Their classification and their relationship to phlogopite-bearing peridotites and kimberlites revisited. Contrib. Mineral. Petrol..

[20] O’Reilly, S. Y. & Griffin, W. L. Mantle metasomatism in *Metasomatism and the Chemical Transformation of Rock* (eds. Harlov, D. E. & Håkon, A.) 471–533 (Springer Berlin Heidelberg, 2013).

[21] Safonov OG, Butvina VG (2016). Indicator reactions of K and Na activities in the upper mantle: Natural mineral assemblages, experimental data, and thermodynamic modeling. Geochem. Intern..

[22] Bédard JH (2013). Parameterizations of calcic clinopyroxene-Melt trace element partition coefficients. Geochem. Geophys. Geosyst..

[23] Pilet S, Baker MB, Stolper EM (2008). Metasomatized lithosphere and the origin of alkaline lavas. Science.

[24] Mayer B, Jung S, Romer RL, Pfänder JA, Klügel A, Pack A, Gröner E (2014). Amphibole in alkaline basalts from intraplate settings: Implications for the petrogenesis of alkaline lavas from the metasomatized lithospheric mantle. Contrib. Mineral. Petrol..

[25] Condamine P, Médard E, Devidal JL (2016). Experimental melting of phlogopite-peridotite in the garnet stability field. Contrib. Mineral. Petrol..

[26] Matchan E, Hergt J, Phillips D, Shee S (2009). The geochemistry petrogenesis and age of an unusual alkaline intrusion in the western Pilbara craton, Western Australia. Lithos.

[27] Demidjuk Z, Turner S, Sandiford M, George R, Foden J, Etheridge M (2007). U-series isotope and geodynamic constraints on mantle melting processes beneath the Newer Volcanic Province in South Australia. Earth Planet. Sci. Lett..

[28] Green DH (1976). Experimental testing of “equilibrium” partial melting of peridotite under water-saturated, high-pressure conditions. Can. Mineral..

[29] Mengel K, Green DH (1989). Stability of amphibole and phlogopite in metasomatized peridotite under water-saturated and water-undersaturated conditions. Austral. J Earth Sci. Spec. Pub..

[30] Perinelli C, Andreozzi GB, Conte AM, Oberti R, Armienti P (2012). Redox state of subcontinental lithospheric mantle and relationships with metasomatism: Insights from spinel peridotites from northern Victoria Land (Antarctica). Contrib. Mineral. Petrol..

[31] Griffin WL, Wass SY, Hollis JD (1984). Ultramafic xenoliths from Bullenmerri and Gnotuk maars, Victoria, Australia: Petrology of a sub-continental crust-mantle transition. J. Petrol..

[32] Boyce J (2013). The Newer Volcanics Province of southeastern Australia: A new classification scheme and distribution map for eruption centres. Aust. J. Earth Sci..

[33] O’Reilly SY, Griffin WL (1988). Mantle metasomatism beneath western Victoria, Australia: I. Metasomatic processes in Cr-diopside lherzolites. Geochem. Cosmochim. Acta.

[34] O’Reilly SY, Zhang M (1995). Geochemical characteristics of lava-field basalts fromeastern Australia and inferred sources, concentrations with subcontinental lithospheric mantle?. Contrib. Mineral. Petrol..

[35] Le Maitre, R. W. *et al.* Igneous Rocks: A Classification and Glossary of Terms, Recommendations of the International Union of Geological Sciences Subcommission of the Systematics of Igneous Rocks. Cambridge University Press, UK (2002).

[36] Brey GP, Köhler TP (1990). Geothermobarometry in four-phase lherzolites II. New thermobarometers, and practical assessment of existing thermobarometers. J. Petrol..

[37] Ballhaus C, Berry R, Green D (1991). High pressure experimental calibration of the olivine-orthopyroxene-spinel oxygen geobarometer: implications for the oxidation state of the upper mantle. Contrib. Mineral. Petrol..

[38] Jianping L, Kornprobst J, Vielzeuf D (1995). An improved experimental calibration of the olivine-spinel geothermometer. Chin. J. Geochem..

[39] Wood BJ (1991). Oxygen barometer of spinel peridotites. Rev. Mineral..

[40] Frost BR (1991). Introduction to oxygen fugacity and its petrologic importance. Rev. Mineral..

[41] Mercier JC, Nicolas A (1975). Textures and fabrics of the upper-mantle peridotites as illustrated by xenoliths from basalts. J. Petrol..

[42] Kinzler RJ, Grove TL (1992). Primary magmas of mid-ocean ridge Basalts 1. Experiments and Methods. J. Geophys. Res..

[43] Walter MJ, Presnall DC (1994). Melting behavior of simplified lherzolite in the system CaO-MgO-Al_2_O_3_-SiO_2_-Na2O from 7 to 35 kbar. J. Petrol..

[44] Kinzler R (1997). Melting of mantle peridotite at pressures approaching the spinel to garnet transition: Application to mid-ocean ridge basalt petrogenesis. J. Geophys. Res..

[45] Walter MJ (1998). Melting of garnet peridotite and the origin of komatiite and depleted lithosphere. J. Petrol..

[46] Herzberg C (2004). Geodynamic information in peridotite petrology. J. Petrol..

[47] Arai S (1994). Characterization of spinel peridotites by olivine-spinel compositional relationships; Review and interpretation. Chem. Geol..

[48] Bonadiman C, Coltorti M, Beccaluva L, Griffin WL, O’Reilly S, Siena F (2011). Metasomatism versus host magma infiltration: a case study of Sal mantle xenoliths, Cape Verde Archipelago. Geol. Soc. Am. Spec. Pap..

[49] Hellebrand E, Snow JE (2003). Deep melting and sodic metasomatism underneath the highly oblique-spreading Lena Trough (Arctic Ocean). Earth Planet. Sci. Lett..

[50] Faccini B, Bonadiman C, Coltorti M, Grégoire M, Siena F (2013). Oceanic material recycled within the Sub-Patagonian Lithospheric Mantle (Cerro del Fraile, Argentina). J. Petrol..

[51] McCarthy A, Müntener O (2019). Evidence for ancient fractional melting, cryptic refertilization and rapid exhumation of Tethyan mantle (Civrari Ophiolite, NW Italy). Contrib. Mineral. Petrol..

[52] Klemme S, O’Neill H (2000). The near-solidus transition from garnet lherzolite to spinel lherzolite. Contrib. Mineral. Petrol..

[53] McDonough WF, Sun SS (1995). The composition of the Earth. Chem. Geol..

[54] Davis FA, Cottrell E, Birner SK, Warren JM, Lopez OG (2017). Revisiting the electron microprobe method of spinel-olivine-orthopyroxene oxybarometry applied to spinel peridotites. Am. Mineral..

[55] Burns RG, Greaves C (1971). Correlations of infrared and Mössbauer site population measurements of actinolites. Am. Mineral..

[56] Dyar MD, Mackwell SM, McGuire AV, Cross LR, Robertson JD (1993). Crystal chemistry of Fe^3+^ and H^+^ in mantle kaersutite: Implications for mantle metasomatism. Amer. Mineral..

[57] Andreozzi GB, Ballirano P, Gianfagna A, Mazziotti-Tagliani S, Pacella A (2009). Structural and spectroscopic characterization of a suite of fibrous amphiboles with high environmental and health relevance from Biancavilla (Sicily, Italy). Am. Mineral..

[58] Bonadiman C, Nazzareni S, Coltorti M, Comodi P, Giuli G, Faccini B (2014). Crystal chemistry of amphiboles: Implications for oxygen fugacity and water activity in lithospheric mantle beneath Victoria Land, Antarctica. Contrib. Mineral. Petrol..

[59] Gunter ME, Dyar MD, Twamley B, Foit FF, Cornelius C (2003). Composition, Fe^3+^/ΣFe, and crystal structure of non-asbestiform and asbestiform amphiboles from Libby, Montana, USA. Am. Mineral..

[60] Hawthorne FC, Oberti R (2007). Amphiboles: crystal chemistry. Rev. Mineral. Geochem..

[61] Bancroft GM, Maddock AG, Burns RG, Strens RGJ (1966). Cation distribution in anthophyllite from Mössbauer and infra-red spectroscopy. Nature.

[62] Bancroft GM, Burns RG, Maddock AG (1967). Determination of cation distribution in the cummingtonite-grunerite series by Mössbauer spectra. Am. Mineral..

[63] Hafner SS, Ghose S (1971). Iron and magnesium distribution in cummingtonites (Fe, Mg_)_7S_i_8_O2_2(OH_)_2. Z. Kristallogr..

[64] Goldman DS, Rossman G (1977). The identification of Fe2+ in the M(4) site of calcic amphiboles. Am. Mineral.

[65] Stroink G, Blaauw C, White CG, Leiper W (1980). Mössbauer characteristics of UICC standard reference asbestos samples. Can. Mineral..

[66] Oberti R, Hawthorne FC, Cannillo E, Cámara F (2007). Long-Range order in amphiboles. Rev. Mineral Geochem..

[67] Hawthorne FC, Oberti R, Harlow GE, Maresch WV, Martin RF, Schumacher JC, Welch MD (2012). IMA Report. Nomenclature of the amphibole supergroup. Am. Mineral..

[68] Brigatti MF, Medici L, Saccani E, Vaccaro C (1996). Crystal chemistry and petrologic significance of Fe^3+^-rich phlogopite from the Tapira carbonatite complex. Braxil. Am. Min..

[69] Johnson KE, Davis AM, Bryndzia LT (1996). Contrasting styles of hydrous metasomatism in the upper mantle: An ion microprobe investigation. Geochim. Cosmochim. Acta.

[70] Adam J, Green T (2006). Trace element partitioning between mica- and amphibole-bearing garnet lherzolite and hydrous basanitic melt: 1. Experimental results and the investigation of controls on partitioning behaviour. Contrib. Mineral. Petrol..

[71] Moine BN, Grégoire M, O’Reilly SY, Sheppard SMF, Cottin JY (2001). High Field Strength Element fractionation in the upper mantle: evidence from amphibole-rich composite mantle xenoliths from the Kerguelen Islands (Indian Ocean). J. Petrol..

[72] Adam J, Turner M, Hauri EK, Turner S (2016). Crystal/melt partitioning of water and other volatiles during the near-solidus melting of mantle peridotite: Comparisons with non-volatile incompatible elements and implications for the generation of intraplate magmatism. Am. Mineral..

[73] Flemetakis S, Klemme S, Stracke A, Genske F, Berndt J, Rohrbach A (2021). Constraining the presence of amphibole and mica in metasomatized mantle sources through halogen partitioning experiments. Lithos.

[74] Grégoire M, Moine BN, O’Reilly SY, Cottin JY, Giret A (2000). Trace element residence and partitioning in mantle xenoliths metasomatized by highly alkaline, silicate- and carbonate-rich melts (Kerguelen Islands, Indian Ocean). J. Petrol..

[75] Krmíček L, Halavínová M, Romer RL, Vašinová Galiová M, Vaculovič T (2014). Phlogopite/matrix, clinopyroxene/matrix and clinopyroxene/ phlogopite trace-element partitioning in a calc-alkaline lamprophyre: new constrains from the Křižanovice minette dyke (Bohemian Massif). J. Geosci..

[76] LaTourette T, Hervig RL, Holloway JR (1995). Trace element partitioning between amphibole, phlogopite, and basanite melt. Earth Planet. Sci. Lett..

[77] Hauri EH, Gaetani GA, Green TH (2006). Partitioning of water during melting of the Earth's upper mantle at H_2_O-undersaturated conditions. Earth Planet. Sci. Lett..

[78] Mattioli GS, Wood B (1988). Magnetite activities across the MgAl_2_O_4_–Fe_3_O_4_ spinel join, with application to thermobarometric estimates of upper mantle oxygen fugacity. Contrib. Mineral. Petrol..

[79] O’Neill HSC, Wall VJ (1987). The olivine—orthopyroxene—spinel oxygen geobarometer, the nickel precipitation curve, and the oxygen fugacity of the earth's upper mantle. J. Petrol..

[80] Miller WGR, Holland TJB, Gibson SA (2016). Garnet and spinel oxybarometers: New internally consistent multi-equilibria models with applications to the oxidation state of the lithospheric mantle. J. Petrol..

[81] Wood BJ, Virgo D (1989). Upper mantle oxidation state: ferric iron contents of lherzolite spinels by ^57^Fe Mössbauer spectroscopy and resultant oxygen fugacities. Geochem. Cosmochim. Acta.

[82] Figueiras J, Waerenborgh JC (1997). Fully oxidized chromite in the Serra Alta (South Portugal) quartzites: Chemical and structural characterization and geological implications. Mineral. Mag..

[83] Stagno V, Fei Y (2020). The Redox boundaries of Earth’s interior. Elements.

[84] Curetti N, Bonadiman C, Compagnoni R, Nodari L, Corazzari I, Pavese A (2018). Phengite megacryst *quasi*-exsolving phlogopite, from Sulu ultra-high pressure metamorphic terrane, Qinglongshan, Donghai County (eastern China): New data for P-T-X conditions during exhumation. Lithos.

[85] Gale JD (2005). GULP: capabilities and prospects. Z. Kristallogr..

[86] O’Reilly SY, Griffin WL (1985). A xenolith-derived geotherm for Southeastern Australia and its geophysical implications. Tectonophysics.

[87] Cull JP (1989). Geothermal models and mantle rheology in Australia. Tectonophysics.

[88] Tesauro M, Kaban MK, Aitken ARA (2020). Thermal and compositional anomalies of the Australian upper mantle from seismic and gravity data. Geochem. Geophys. Geosyst..

[89] Tesauro M, Kaban MK, Petrunin AG, Aitken ARA (2020). Strength variations of the Australian continent: Effects of temperature, strain rate, and rheological changes. Glob. Planet. Change.

[90] Dovesi, R. *et al.* Quantum‐mechanical condensed matter simulations with CRYSTAL. *Wiley Interdisciplinary Reviews: Computational Molecular Science* **8**, e1360. 10.1002/wcms.1360 (2018).

[91] Huang Y, Nakatani T, Nakamura M, McCammon C (2019). Saline aqueous fluid circulation in mantle wedge inferred from olivine wetting properties. Nat. Commun..

[92] Foley SF (1992). Petrological characterization of the source components of potassic magmas: Geochemical and experimental constraints. Lithos.

[93] Aulbach S, Woodland AB, Stern RA, Vasilyev P, Heaman LM, Viljoen KS (2019). Evidence for a dominantly reducing Archaean ambient mantle from two redox proxies, and low oxygen fugacity of deeply subducted oceanic crust. Sci. Rep..

[94] Lu J, Griffin WL, Tilhac R, Xiong Q, Zheng J, O’Reilly SY (2018). Tracking deep lithospheric events with garnet-websterite xenoliths from southeastern Australia. J. Petrol..

[95] Wilkins D, Gouramanis C, De Deckker P, Fifield LK, Olley J (2013). Holocene lake-level fluctuations in Lakes Keilambete and Gnotuk, southwestern Victoria, Australia. The Holocene.

[96] Lachance GR, Traill RJ (1966). Practical solution to the matrix problem in X-ray analysis. Can. J. Spectros..

[97] Zhang C, Koepke J, Wang L-X, Wolff PE, Wilke S, Stechern A, Almeev RR, Holtz FA (2016). Practical Method for Accurate measurement of trace level fluorine in Mg- and Fe-bearing minerals and glasses using electron probe microanalysis. Geostand. Geoanal. Res..

[98] Oberti R, Hawthorne FC, Ungaretti L, Cannillo E (1995). Al disorder in amphiboles from mantle peridotites. Can. Mineral..

[99] Oberti R, Vannucci R, Zanetti A, Tiepolo M, Brumm RC (2000). A crystal chemical re-evaluation of amphibole/melt and amphibole/clinopyroxene D_Ti_ values in petrogenetic studies. Am. Mineral..

[100] Rüffer R, Chumakov AI (1996). Nuclear-resonance beamline at ESRF. Hyperfine Interact..

[101] Potapkin V, Chumakov AI, Smirnov GV, Celse J-P, Rüffer R, McCammon C, Dubrovinsky L (2012). The ^57^Fe synchrotron Mössbauer source at the ESRF. J. Synchrotron. Radiat..

[102] Prescher C, McCammon C, Dubrovinsky L (2012). MossA: a program for analyzing energy-domain Mössbauer spectra from conventional and synchrotron sources. J. Appl. Crystallogr..

[103] Leitner J, Sedmidubský D, Chuchvalec P (2002). Prediction of heat capacities of solid binary oxides from group contribution method. Ceramics-Silikáty.

[104] Shulman LM (2004). The heat capacity of water ice in interstellar or interplanetary conditions. Astron. Astrophys..

[105] Ferse A (2013). Numerical values of individual activity coefficients of single-ion species in concentrated aqueous electrolyte solutions and the attempt of a qualitative interpretation on a model of electrostatic interaction. J. Solid State Electrochem..

[106] Merli M, Sciascia L, Pavese A, Diella V (2015). Modelling of thermo-chemical properties over the sub-solidus MgO–FeO binary, as a function of iron spin configuration, composition and temperature. Phys. Chem. Minerals.

[107] Catlow, C. R. A. CATLOW library - collection of potentials basedaround the Catlow oxygen-oxygen potential. www.ucl.ac.uk/klmc/Potentials/Library/catlow.lib (1992)

[108] Zhao Y, Truhlar DG (2008). Density functionals with broad applicability in chemistry. Acc. Chem. Res..

[109] Merli M, Pavese A (2018). Electron-density critical points analysis and catastrophe theory to forecast structure instability in periodic solid. Acta Cryst. A.

